# Cellular mechanisms of oligoclonal vascular smooth muscle cell expansion in cardiovascular disease

**DOI:** 10.1093/cvr/cvac138

**Published:** 2022-08-22

**Authors:** Matt D Worssam, Jordi Lambert, Sebnem Oc, James C K Taylor, Annabel L Taylor, Lina Dobnikar, Joel Chappell, Jennifer L Harman, Nichola L Figg, Alison Finigan, Kirsty Foote, Anna K Uryga, Martin R Bennett, Mikhail Spivakov, Helle F Jørgensen

**Affiliations:** Section of Cardiorespiratory Medicine, Department of Medicine, University of Cambridge, Papworth Road, Cambridge Biomedical Campus, Cambridge CB2 0BB, UK; Section of Cardiorespiratory Medicine, Department of Medicine, University of Cambridge, Papworth Road, Cambridge Biomedical Campus, Cambridge CB2 0BB, UK; Section of Cardiorespiratory Medicine, Department of Medicine, University of Cambridge, Papworth Road, Cambridge Biomedical Campus, Cambridge CB2 0BB, UK; Section of Cardiorespiratory Medicine, Department of Medicine, University of Cambridge, Papworth Road, Cambridge Biomedical Campus, Cambridge CB2 0BB, UK; Section of Cardiorespiratory Medicine, Department of Medicine, University of Cambridge, Papworth Road, Cambridge Biomedical Campus, Cambridge CB2 0BB, UK; Section of Cardiorespiratory Medicine, Department of Medicine, University of Cambridge, Papworth Road, Cambridge Biomedical Campus, Cambridge CB2 0BB, UK; Babraham Institute, Cambridge, UK; Section of Cardiorespiratory Medicine, Department of Medicine, University of Cambridge, Papworth Road, Cambridge Biomedical Campus, Cambridge CB2 0BB, UK; Section of Cardiorespiratory Medicine, Department of Medicine, University of Cambridge, Papworth Road, Cambridge Biomedical Campus, Cambridge CB2 0BB, UK; Section of Cardiorespiratory Medicine, Department of Medicine, University of Cambridge, Papworth Road, Cambridge Biomedical Campus, Cambridge CB2 0BB, UK; Section of Cardiorespiratory Medicine, Department of Medicine, University of Cambridge, Papworth Road, Cambridge Biomedical Campus, Cambridge CB2 0BB, UK; Section of Cardiorespiratory Medicine, Department of Medicine, University of Cambridge, Papworth Road, Cambridge Biomedical Campus, Cambridge CB2 0BB, UK; Section of Cardiorespiratory Medicine, Department of Medicine, University of Cambridge, Papworth Road, Cambridge Biomedical Campus, Cambridge CB2 0BB, UK; Section of Cardiorespiratory Medicine, Department of Medicine, University of Cambridge, Papworth Road, Cambridge Biomedical Campus, Cambridge CB2 0BB, UK; Functional Gene Control Group, MRC London Institute of Medical Sciences, London, UK; Institute of Clinical Sciences, Imperial College London, London, UK; Section of Cardiorespiratory Medicine, Department of Medicine, University of Cambridge, Papworth Road, Cambridge Biomedical Campus, Cambridge CB2 0BB, UK

**Keywords:** Vascular smooth muscle cells, Lineage tracing, Single-cell transcriptomics, Clonal dynamics

## Abstract

**Aims:**

Quiescent, differentiated adult vascular smooth muscle cells (VSMCs) can be induced to proliferate and switch phenotype. Such plasticity underlies blood vessel homeostasis and contributes to vascular disease development. Oligoclonal VSMC contribution is a hallmark of end-stage vascular disease. Here, we aim to understand cellular mechanisms underpinning generation of this VSMC oligoclonality.

**Methods and results:**

We investigate the dynamics of VSMC clone formation using confocal microscopy and single-cell transcriptomics in VSMC-lineage-traced animal models. We find that activation of medial VSMC proliferation occurs at low frequency after vascular injury and that only a subset of expanding clones migrate, which together drives formation of oligoclonal neointimal lesions. VSMC contribution in small atherosclerotic lesions is typically from one or two clones, similar to observations in mature lesions. Low frequency (<0.1%) of clonal VSMC proliferation is also observed *in vitro*. Single-cell RNA-sequencing revealed progressive cell state changes across a contiguous VSMC population at onset of injury-induced proliferation. Proliferating VSMCs mapped selectively to one of two distinct trajectories and were associated with cells showing extensive phenotypic switching. A proliferation-associated transitory state shared pronounced similarities with atypical SCA1+ VSMCs from uninjured mouse arteries and VSMCs in healthy human aorta. We show functionally that clonal expansion of SCA1+ VSMCs from healthy arteries occurs at higher rate and frequency compared with SCA1− cells.

**Conclusion:**

Our data suggest that activation of proliferation at low frequency is a general, cell-intrinsic feature of VSMCs. We show that rare VSMCs in healthy arteries display VSMC phenotypic switching akin to that observed in pathological vessel remodelling and that this is a conserved feature of mouse and human healthy arteries. The increased proliferation of modulated VSMCs from healthy arteries suggests that these cells respond more readily to disease-inducing cues and could drive oligoclonal VSMC expansion.

## Graphical Abstract

**Graphical Abstract:**
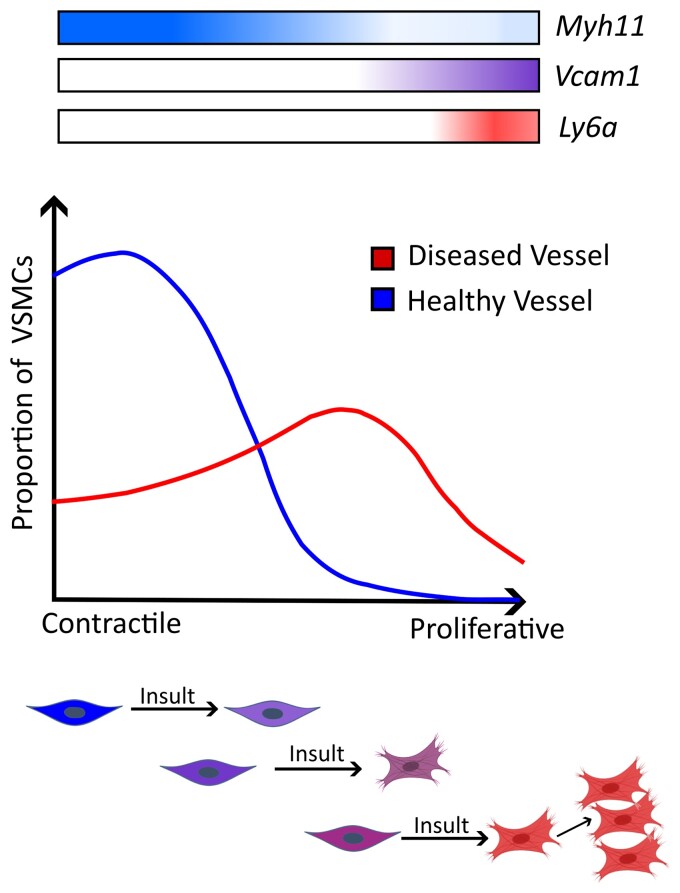

**See the editorial comment for this article ‘Smooth muscle cell oligoclonality in vascular disease: same origin, different destinies’, by L. Matic et al., https://doi.org/10.1093/cvr/cvad050.**

## 1. Introduction

Vascular smooth muscle cells (VSMCs) form the medial layer of major arteries and their contractility controls vessel tone. In healthy adult arteries, VSMCs are quiescent but retain remarkable plasticity.^1^ In response to vascular inflammation and injury, VSMCs can exit from quiescence, become migratory and downregulate components of the smooth muscle-contractile machinery (such as *MYH11* and *ACTA2*), while increasing the expression of extracellular matrix components (e.g. matrix gla protein, MGP, and collagen).^2^ VSMC plasticity, termed phenotypic switching, ensures tissue homeostasis and enables physiological vessel remodelling. However, dysregulated VSMCs cause neointimal growth after surgical intervention and generate the majority of cells in atherosclerotic lesions. Characterization of VSMC-lineage-labelled plaque cells has shown that VSMCs both generate the stabilizing fibrous cap and cells in the lesion core that have characteristics of mesenchymal cells, phagocytes, and osteochondrocytes, thought to destabilize lesions.^1,3–5^ Deciphering how VSMC plasticity is regulated will therefore help understand the functional role of VSMC-derived cells in vascular health and disease.

Multi-colour lineage-tracing studies in mouse atherosclerosis models showed that VSMC-derived plaque cells are oligoclonal, generated from very few pre-existing VSMCs.^6–10^ Similar clonal VSMC contribution has also been observed in aortic dissection and after injury,^6,11,12^ and the clonal progeny of individual VSMCs can generate the diverse phenotypes observed in atherosclerotic lesions.^6,13^ The oligoclonal nature of VSMCs in disease is different from what has been reported in development^14^ and appears at odds with observations suggesting widespread medial proliferation after injury.^15–17^ These discrepancies call for analysis of whether lesion clonality of VSMC-derived cells results from rare activation of VSMCs, or if this is due to differential survival of VSMC clones following a general proliferative response (reviewed in Worssam and Jørrgensen^18^). Identifying the cellular mechanisms underlying clonality is required to understand how VSMC proliferation is controlled and could be manipulated.

We here measured VSMC clone dynamics in cardiovascular disease models with VSMC phenotypic switching *in vitro* and *in vivo*. This was combined with scRNA-seq analysis, demonstrating that VSMCs adopt a continuum of phenotypic states after vessel injury. Our analysis suggests that proliferation does not result from activation of a dedicated progenitor population, but that cells showing pronounced phenotypic switching along this continuum have increased proliferative capacity. In mouse, pre-proliferative VSMCs express SCA1 and exist at low abundancy in healthy arteries. VSMCs with characteristics of mouse SCA1+ cells were also found in healthy human arteries, suggesting that a conserved mechanism may underlie development of human vascular disease.

## 2. Methods

### 2.1 Human tissue

Anonymized human arteries from patients undergoing carotid endarterectomy [plaques; 50-year-old female (50F), 75-year-old male (75M), 74M, 54M] or coronary artery bypass/valve replacement (normal aorta; 52M, 63F, 42M, 60M, 85M, 74M) were obtained under informed consent and approved by the Cambridge or Huntingdon Research Ethical Committee. The experiments with human samples conform to the principles outlined in the Declaration of Helsinki.

### 2.2 Animals and procedures

Animal experiments were approved by the local ethics committee and performed according to UK Home Office regulation under project licence P452C9545. All alleles have been described previously; Myh11-CreERt2 is a Y-linked transgene that confers expression of a tamoxifen-inducible Cre recombinase in smooth muscle cells,^19,20^ Rosa26-Confetti,^21^ and Rosa26-EYFP^22^ are Cre-recombination reporter alleles, KI67-RFP is an insertion in the Mki67 locus resulting in expression of a KI67-RFP fusion protein^23^ and the mutant Apoe allele sensitizes mice to high fat diet (HFD)-induced atherosclerosis development.^24^ Myh11-CreERt2 is Y-linked, so all VSMC-lineage-tracing experiments were performed using males. VSMC-lineage labelling by tamoxifen administration was followed by a washout period before animals were subjected to carotid ligation surgery under anaesthesia (isoflourane by inhalation; 2.5–3%, 1.5 L/min for induction, maintained at 1.5%) or fed a HFD containing 21% fat and 0.2% cholesterol (Special Diets Services) as described.^6^ Animals were euthanized by cervical dislocation or increasing CO_2_ concentration. Perfusion fixation was not performed to preserve expression of Confetti-reporter proteins.

### 2.3 Tissue processing

Mouse arteries were fixed after dissection, stained with 4′, 6-diamidino-2-phenylindole (DAPI), cleared, and mounted in RapiClear 1.52 (whole mount imaging). Fixed arteries were cryopreserved, frozen in optimal cutting temperature compound (Tissue-Tek), and cryosectioned into thin (14 μm) or thick (100 μm) sections. Human arteries were formaldehyde-fixed and paraffin-embedded (FFPE) before sectioning (4 μm) for staining. Single-cell suspensions were generated by Collagenase IV (Invitrogen) and Elastase (Worthington) digestion.

### 2.4 *In vitro* proliferation assays

The adventitial layer was removed from aortas of Myh11-Confetti animals and after overnight incubation in Opti-MEM, tissue sections for explant analysis (1 mm^2^) were cut, pinched with sharp forceps to create an internal injury site and embedded in Matrigel in an 8-well chamber slide (Ibidi). Explanted sections were fixed before or after 8 days of culture and mounted in RapiClear 1.47 (Sunjin Lab).

Enzyme digested medial cells from VSMC-lineage labelled animals and wild-type animals were mixed (1:3 ratio for Myh11-Confetti; 1:9 for fluorescence-activated cell sorting (FACS)-isolated SCA1+EYFP+ or SCA1−EYFP+ cells). A total of 5000 cells were seeded per well of a 96-well imaging plate (CellCarrier-96 Ultra, Perkin Elmer) and imaged using an Opera Phenix high-content screening system (Perkin Elmer). Image analysis was done using Harmony software (Perkin Elmer) and Fiji.^25^ Patches were defined as an area with three or more contiguous cells of same reporter colour and patch area determined by generating a mask using thresholding after enhancing local contrast (CLAHE).^26^

### 2.5 Analysis of tissue sections

Cryosections were permeabilized, blocked, and incubated with primary antibodies (see Supplementary material online, *Table S1*) or isotype controls followed by Alexa-647 conjugated secondary antibodies, and nuclei counterstained with DAPI before mounting in RapiClear 1.52 (Sunjin Lab). FFPE sections were dewaxed, antigen retrieval-treated, and blocked before staining with first anti-FBLN2 or isotype control, visualized with HRP-conjugated anti-Rabbit (Cell Signalling Technology, 8114) and DAB peroxidase substrate (SignalStain), and subsequently with anti-αSMA (DAKO, M0851), visualized with biotin-coupled anti-Mouse (DAKO, E0433), and Vectastain avidin-coupled alkaline phosphatase with Blue AP substrate solution (Vector Labs) before mounting in VectaMount mounting media (Vector Labs). RNA *in situ* hybridization of Hs-LUM-C1 probes coupled to Opal™ 690 was performed using RNA Scope® Multiplex Fluorescent v2 kits (ACD), according to the manufacturer’s instructions and imaged using a ZEISS Axioscan slide scanner.

### 2.6 Confocal microscopy and image analysis

Confocal imaging was done using a Leica SP8 microscope with sequential, resonant, tile scan mode with Z-compensation (laser power) to normalize fluorescent protein intensity in thick specimens. Laser lines and detector settings are described in Supplementary material online, *Table S2*. Tile stitching was done using the mosaic merge function in LASX (Leica), and subsequent image analysis in Imaris (9.0.2). Scoring of VSMC patches and bulged regions in injured arteries and VSMC plaque contribution is described in the Supplementary material online. Arteries with poor image quality (tissue damage or autofluorescence, total of eight arteries) or arteries analysed more than 10 days after injury that did not show any sign of reaction to surgery (e.g. adventitial expansion, three arteries) were excluded from analysis.

### 2.7 FACS, flow cytometry, image stream

Single-cell suspensions were blocked with TruStain FcX anti-mouse CD16/32 antibody (Biolegend) and incubated with primary or isotype control antibody (see Supplementary material online, *Table S1*) for 15 min at room temperature followed by secondary antibody where needed. Intracellular targets were stained using the Foxp3 staining buffer set (eBioscience) and phalloidin-iFluor™ 350 was included with secondary antibodies. Stained cells were sorted (BD FACSAria™ III, BD Bioscience) or analysed by flow cytometry (Accuri C6 or BD Fortessa, BD Bioscience) or Imagestream (Amnis® ImageStream®^X^Mk II, Luminex). Flow cytometry compensation used single stained samples and gates were defined based on samples stained with control antiserum and wild-type cells.

### 2.8 scRNA-seq analysis

Single-cell suspensions of arterial cells from VSMC-lineage-labelled animals (see Supplementary material online) were loaded onto the Chromium system (10× Genomics) or processed using the Smart-seq2 protocol^27^ as described^13^ with addition of ERCC spike controls (diluted 1:80 000 000, Invitrogen). Pooled cDNA libraries were sequenced and reads were aligned to the GRCm38 mouse genome (D7, Smartseq-2) or a custom genome based on the GRCm38 mouse genome, including the coding sequence of EYFP^22^ (D5-EYFP, D5-all arterial cells). Quality control (QC), filtering, normalization, and gene expression analysis were performed using the CRAN R package *Seurat* v.3.1.2^28,29^ in R v.3.6.2. Normalization was performed using *SCTransform*^30^ v.0.2.1. Cell cluster marker genes identification was done by differential expression testing with Wilcoxon rank sum test (FindAllMarkers, log fold-change >0.5, adjusted *P*-value < 0.05) and visualized using the DoHeatmap function in *Seurat*. Data integration (mouse D7 Smart-seq2 and D7 10× datasets, or mouse D5-all arterial cells and SCA1 Smart-seq2 datasets) was done using *Seurat* v.3.2.1^28^ following log normalization. Trajectory inference was performed using the Bioconductor R package *slingshot* v.1.4.0^31^ or partition-based graph abstraction (PAGA) in *ScanPy.*^32^ Genes showing differential expression along pseudotime were identified using the CRAN R package *gam* v.1.16.1 and hierarchically clustered according to Pearson correlation distance using the CRAN R package *pheatmap* v.1.0.12, and the number of gene clades determined by CRAN R package *factoextra* v.1.0.7. Gene ontology (GO) term analysis was performed with the enrichGO and compareCluster functions implemented in Bioconductor R package *clusterProfiler* v.3.14.3^33^ using the Bioconductor R package *org.Mm.eg.db* v.3.10.0. with multiple testing correction (Benjamini–Hochberg) and *P*-value and *q*-value thresholds of 0.05. The summarized expression level of gene subsets was computed from the first principal components across cells in the respective dataset and the Module Score calculated using the AddModuleScore function in *Seurat*. The gene list of the VSMC response signature was obtained from Dobnikar *et al*.^13^ Path1-induced genes included Clades 1, 2, 3, 4, 8 (Path1).

### 2.9 Statistical analysis

Statistical analysis was performed in *R* or *GraphPad Prism v7*. Statistical significance tests, selected after assessing normality (Shapiro–Wilk) and equal variance (Bartlett or Levine) are indicated in figure legends. Local regression analysis was used to fit a LOESS curve for patch number analysis. To assess the statistical significance of the association between Confetti, CD45, and KI67 protein expression and bulged or non-bulged artery status, Fisher’s Exact test was used on raw count data of positive cells for each marker in each region. To assess statistical significance of SCA1 expression status in the clonal proliferation assay, a generalized linear model was fitted for patch number, whereas multiple linear regression was used for patch area, as the data showed equal variance and linearity and the residuals were approximately normally distributed.

## 3. Results

### 3.1 Proliferation is restricted to a small number of VSMCs after vascular injury

**Figure 1:**
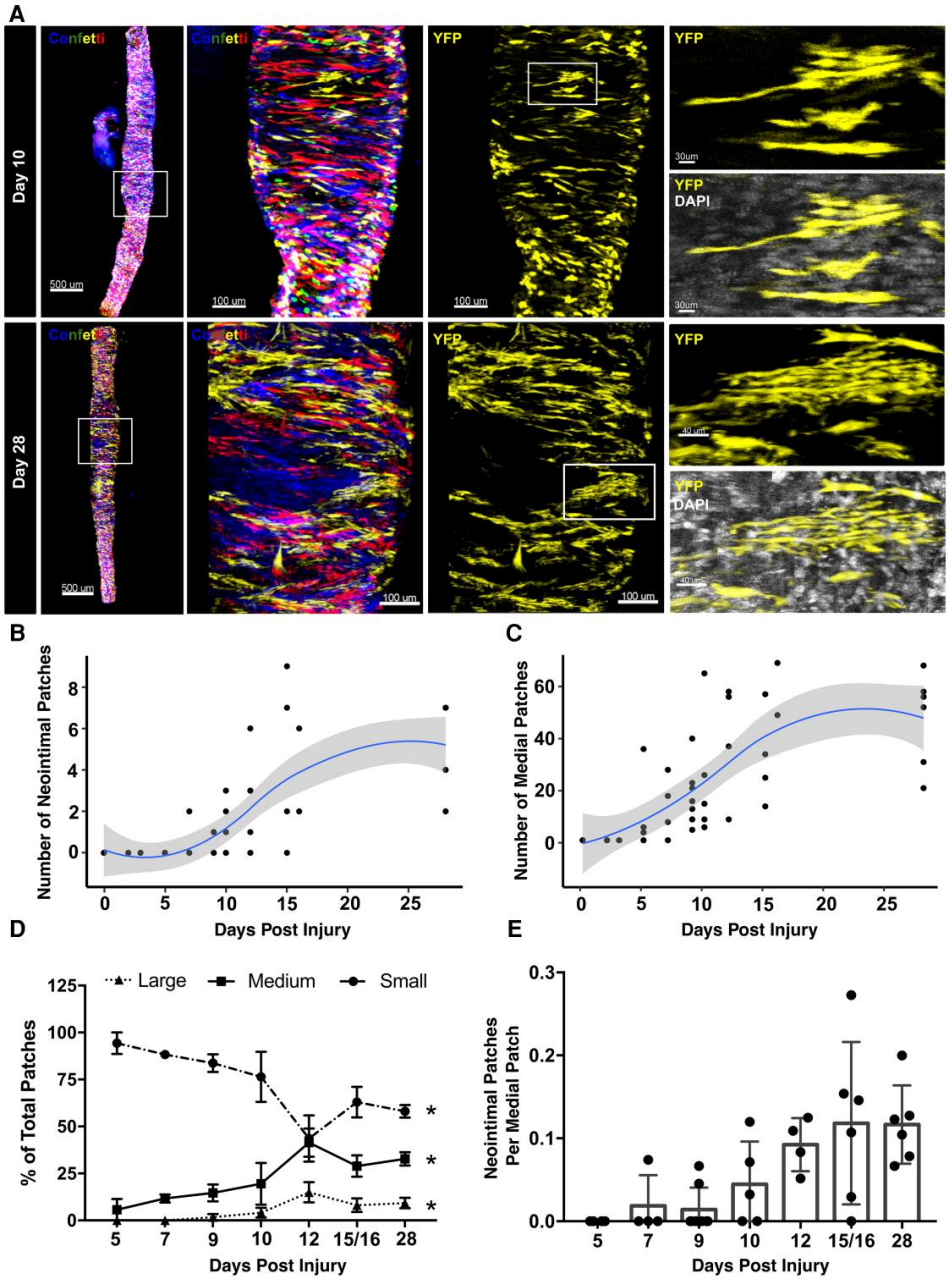
Analysis of VSMC clone dynamics after vascular injury. (*A*) Representative whole-mounted left carotid arteries 10 (top) and 28 days (lower) after ligation surgery in VSMC-lineage-labelled Myh11-Confetti animals (max. projections of confocal *Z*-stacks). The first two panels show signals for all Confetti proteins (CFP: blue, RFP: red, YFP: yellow, GFP: green) in the whole vessel (scale bar = 500 μm) and a magnified view of the boxed region (scale bar = 100 μm). Third panels only show signals for the yellow fluorescent protein (YFP, yellow) of the Confetti reporter for the magnified arterial segment. Far-right panels show zooms illustrating VSMC singlets and either a small (Day 10, scale bar = 30 μm) or a large VSMC patch (Day 28, scale bar = 40 μm). For zoomed panels, YFP (yellow) is shown alone (top) or along DAPI signals (white). (*B–D*) Quantification of neointimal (*B*) or medial VSMC patch numbers (*C*) and the size distribution of medial patches (*D*) over time after injury in a total of 43 arteries. (*B* and *C*) Dots show values for individual arteries, blue line shows local polynomial regression (LOESS), and grey area represents the 95% confidence interval. (*D*) The fraction of small (dots), medium (squares), and large medial VSMC patches (triangles) are shown for each analysis time point (mean and S.E.M.). Asterisks indicate statistically significant change over time for all size groups (*P* < 0.05, Kruskal–Wallis). (*E*) Bar chart showing the number of neointimal patches as a fraction of medial patches for each artery at indicated time after surgery. Values for individual arteries, mean, and standard deviation are shown.

To understand whether the observed oligoclonal VSMC contribution in vascular disease^6–9^ results from induction of proliferation at low frequency or from clonal competition following general activation of VSMC proliferation,^34^ we studied the dynamics of VSMC clones in lineage-traced animals. Carotid ligation injury was used to induce acute VSMC proliferation with reproducible kinetics.^15,35^ Prior to injury, stable VSMC-specific expression of GFP, RFP, CFP, or YFP was induced in a random manner in Myh11-CreERt2/Rosa26-Confetti + (Myh11-Confetti) mice and clone development observed by scoring monochromatic VSMC patches over time (*Figure 1A*). Importantly, we previously demonstrated an oligoclonal VSMC contribution in neointimal lesions 28 days after surgery in this model,^6^ similar to that observed in atherosclerosis models.^6–9^ Consistent with previous findings,^15^ neointimal lesions were found in 15/16 animals analysed 12 or more days after surgery, but infrequently before Day 10 (see Supplementary material online, *Table S3* and *Figure S1*). At all time points, neointimal lesions were formed by very few monochromatic VSMC patches (*Figure 1B*), similar to our reports at Day 28.^6^ Contiguous monochromatic patches were also identified in the medial layer of 36/43 injured arteries, but not in control animals (*Figure 1C*). Small VSMC patches in the medial layer were not observed prior to Day 5 (D5), where medial clones were detected in two out of three arteries (*Figure 1C* and Supplementary material online, *Table S3*). Formation of medial VSMC patches shortly after injury is consistent with previous observations of proliferation in the medial layer prior to intimal VSMC invasion.^15^ Quantification demonstrated a gradual increase in both medial patch number and size over time, with a possible plateau after 2 weeks (*Figure 1B–D*). The number of intimal VSMC clones was ∼10-fold lower than medial patch numbers, suggesting that only a subset of VSMCs that activate proliferation contribute to neointimal lesion formation (*Figure 1B, C, and E*). In general, the medial layer showed a largely mosaic pattern of the VSMC-lineage reporters, highlighting that the majority of VSMCs do not proliferate.

**Figure 2:**
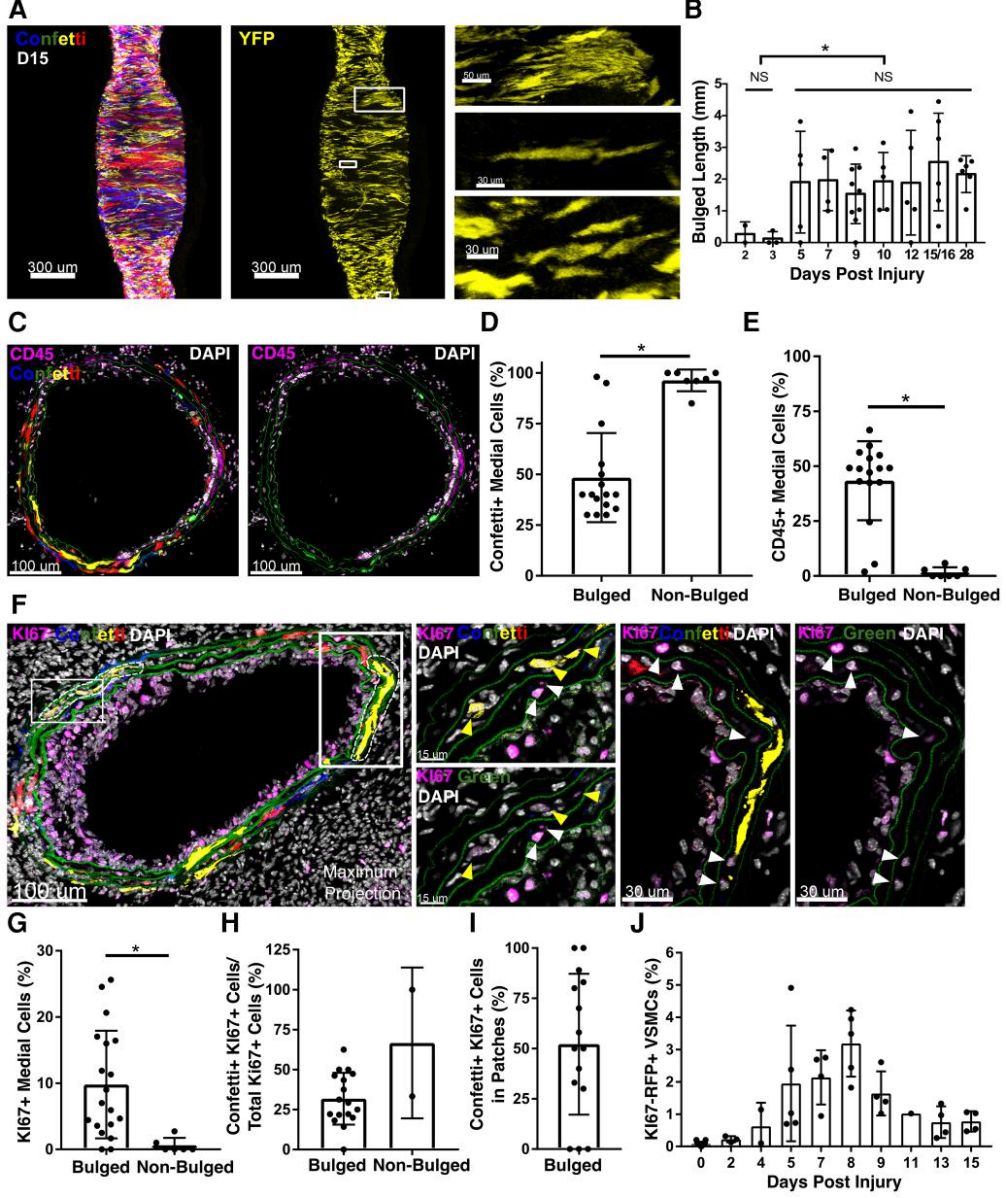
Low proliferation frequency of medial VSMCs. (*A*) Confocal image (max. projection of *Z*-stack) of a Myh11-Confetti carotid artery 15 days (D15) after ligation, providing a representative example showing co-localization of VSMC patches with ‘bulged’ regions with increased vessel diameter. Signals for all Confetti reporters (CFP: blue, RFP: red, YFP: yellow, GFP: green, left panel) or only yellow fluorescent protein (YFP, yellow, middle and right panels) are shown (scale bar = 300 μm). Right panels show magnified views of boxed regions with examples of a patch (top) or a non-patch VSMC singlet (middle) in a bulged region, and several VSMC singlets in an un-remodeled, non-bulged arterial segment (lower). Scale bar = 50 μm (top) or 30 μm. (*B*) Length of bulged region in carotid arteries analysed at indicated times after ligation. Dots show bulge length in individual arteries (43 total), bars show mean and error bars standard deviation. Asterisk indicates significant difference between D2/3 and D5–28 groups (*P* < 0.05, Welch’s *t*-test); NS indicates no significant difference within groups (D2/3: Welch’s *t*-test; D5–D28 one-way ANOVA). (*C*) Representative confocal images of CD45 immunostaining in cryosections from an injured carotid artery (5–7 days post-injury). Signals show CD45 (magenta), DAPI (white), and either all Confetti channels (CFP: blue, RFP: red, YFP: yellow, GFP: green, left) or only the green channel (to show laminal autofluorescence, right). (*D* and *E*) Quantification of the percentage of all DAPI+ medial cells that express Confetti proteins (*D*) or CD45 (*E*) in bulged and non-bulged regions. Dots show data per section (*n* = 3 animals, eight sections per animal). Asterisk indicates *P* < 0.05, Fisher’s Exact Test. *F*, representative confocal images of KI67 immunostaining in cryosections from an injured carotid artery 5–7 days post-injury. Left-hand panel shows a max. projection of the whole immunostained cryosection. Dashed regions indicate VSMC patches. Scale bar = 100 μm. Magnified views of single *Z*-planes for boxed regions are shown in central (scale bar = 15 μm) and right-hand panels (scale bar = 30 μm). Signals show KI67 (magenta), DAPI (white), and either all Confetti channels (CFP: blue, RFP: red, YFP: yellow, GFP: green) or only the green channel (to show laminal autofluorescence). Yellow arrows point to Confetti+KI67+ cells, white arrows show Confetti-KI67+ cells. (*G–I*) Quantification of the percentage of medial cells that express KI67 (*G*), the percentage of medial KI67+ cells that express Confetti (*H*) and the percentage of Confetti+I67+medial cells within patches, in bulged and non-bulged regions of injured carotid arteries (*G* and *H*), or only bulged regions (*I*). Dots show values per section (*n* = 3 animals, average of eight sections analysed per animal). Asterisk indicates *P* < 0.05, Fisher’s Exact Test. (*J*) The fraction of VSMCs (EYFP+) cells expressing the KI67/RFP reporter in flow cytometric analysis of VSMCs from Myh11-EYFP-KI67/RFP animals. Dots show values for individual arteries (32 total), bars represent mean and error bars standard deviation.

Carotid ligation-induced medial VSMC patches were located at arterial segments that had increased diameter (*Figure 2A*). Such remodelled, or ‘bulged’, segments were unique to injured vessels, distributed uniformly along all arteries containing VSMC patches, and also observed in 2/5 arteries prior to D5 (see Supplementary material online, *Figure S1E* and *Table S3*). No apparent change in length was seen after D5 (*Figure 2B*). We observed areas of disorganized cellular arrangement and reduced cell density (*Figure2A* and *B* and Supplementary material online, *Figure S1C–F*) in bulged regions, which is consistent with the observation of cell death after surgery in this model.^36^ Notably, at all time points, a large number of ‘singlet’ VSMCs persisted (90% at Day 28), even within the bulged regions (*Figure 2A* and Supplementary material online, *Figure S1*), demonstrating that despite sharing the signalling environment, not all VSMCs in remodelled arterial regions undergo clonal expansion. This analysis suggests that most VSMCs remain quiescent after injury, and that proliferation is induced in a small number of cells in regions of arterial remodelling.

Previous studies detected BrdU in >20% of medial cells after 5 days of continuous BrdU administration.^15^ To investigate how such widespread medial BrdU incorporation aligns with the infrequent induction of VSMC proliferation suggested by our clonal analysis, we analysed sections from lineage-labelled animals 5–7 days after ligation (*Figure 2C–I*). Immunostaining demonstrated a substantial influx of CD45+ cells to the medial layer of bulged regions (*Figure2C* and *E*) as previously reported,^37^ with a reciprocal decrease in frequency of Confetti+ cells (*Figure 2D*). The proportion of medial cells expressing KI67 varied between animals and was significantly increased in bulged compared with non-bulged regions (*Figure2F* and *G*). On average only 1/3 of the KI67 signal mapped to Confetti+ cells, demonstrating that the majority of proliferating medial cells is not of VSMC origin, but could be infiltrating immune cells (*Figure 2H*). KI67+Confetti+ medial cells were mainly located in monochromatic patches (*Figure 2I*), consistent with clonal VSMC expansion following infrequent activation of proliferation. Further confirming a low VSMC proliferation frequency, the proportion of VSMC-lineage-traced cells expressing a KI67-driven reporter remained low (<5%) throughout (*Figure 2J*). These results suggest that proliferation of non-VSMCs and clonal expansion of VSMCs at low frequency account for the observed frequency of BrdU+ cells.

Taken together, we did not find evidence of a transient surge of VSMC proliferation or VSMC patch number at early time points to indicate clonal competition. Instead, our data are consistent with a model where proliferation is induced in a small number of VSMCs that undergo clonal expansion. Interestingly, our data also suggest that not all medial VSMC clones migrate across the inner elastic lamina to form oligoclonal neointimal lesions.

### 3.2 VSMC investment in atherosclerotic plaques mimics the injury-response

**Figure 3:**
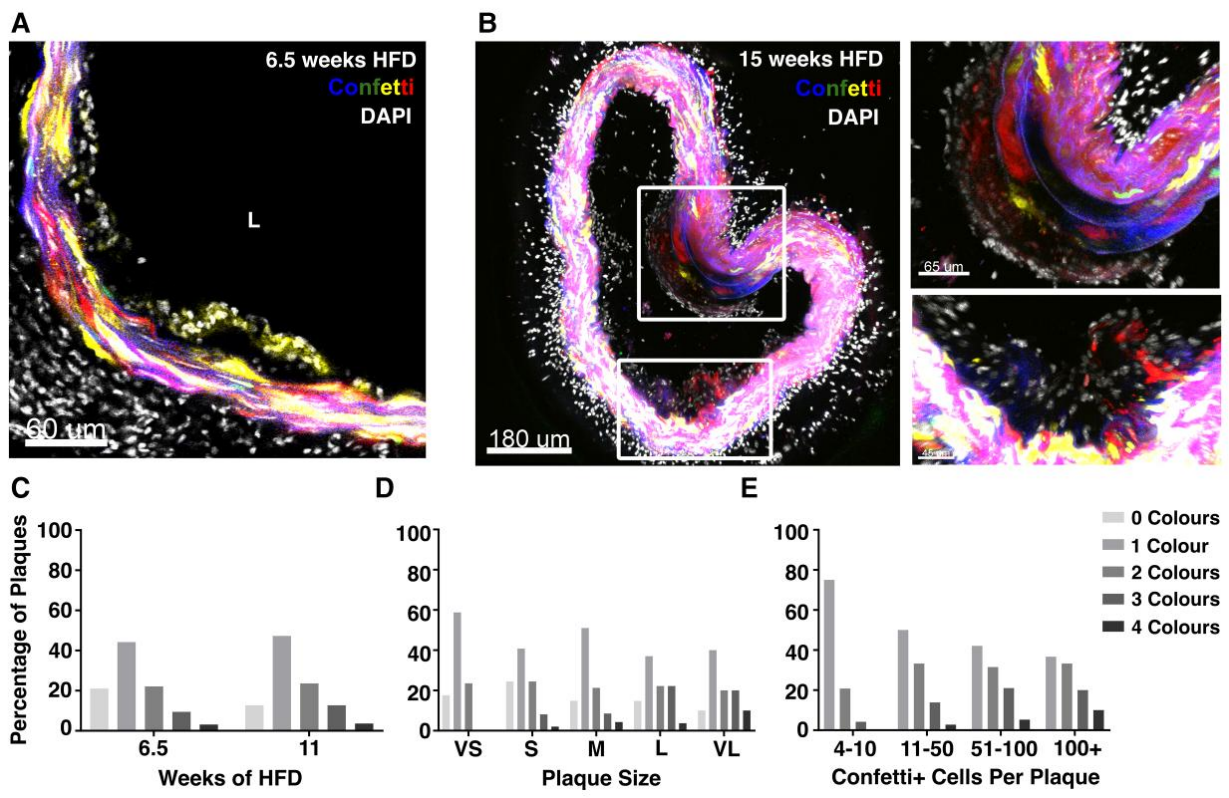
Early atherosclerotic plaques show clonal VSMC investment. (*A* and *B*) Representative confocal images showing VSMC contribution to early lesions in arterial cryosections of high fat diet (HFD)-fed lineage-labelled Myh11-Confetti-Apoe animals (*n* = 9, 6.5–15 weeks of high fat diet, HFD). Confetti (CFP: blue, RFP: red, YFP: yellow, GFP: green) and DAPI (white) signals are shown. (*A*) Single *Z*-plane, 6.5 weeks HFD, scale bar = 60 μm. (*B*) Maximum projection, 15 weeks HFD. Magnified views of boxed regions are shown on the right. Scale bar = 180 μm (left), 65 μm (top right), or 45 μm (lower right). (*C–E*) Confetti colour distribution in lesions from Myh11-Confetti-Apoe animals. Bar graphs show the percentage of plaques where 0, 1, 2, 3, 4 Confetti colours were detected, stratified by time of HFD (*C*, 6.5 weeks: total of 95 lesions in four animals or 11 weeks: total of 55 lesions in three animals), plaque size from very small (VS) to very large (VL). (*D*, VS: 17 lesions total, S: 49, M: 47, L: 27, VL: 10) and overall number of lineage-labelled VSMCs in the plaque (*E*, 4–10 Confetti+ cells: 24 lesions total, 11–50: 36, 51–100: 19, >100: 30).

Next, we used the Confetti model in Apoe^–/–^ animals (Myh11-Confetti-Apoe) to assess whether infrequent activation of VSMC proliferation also underlies oligoclonal VSMC contribution observed in HFD-induced atherosclerosis.^6–9^ Contiguous regions with VSMCs of a single colour were evident at early stages of lesion formation in animals fed a HFD for 6.5–15 weeks (*Figure3A* and *B*).^7^ Quantification showed increasing plaque size in HFD-fed animals at 11 compared with 6.5 weeks, as expected (see Supplementary material online, *Figure S2A*). Lineage-labelled cells were observed in a subset of lesions at both time points, but the frequency of plaques with VSMC investment and the number of VSMCs per plaque increased with duration of HFD (*Figure3C* and Supplementary material online, *Figure S2B*). More than 40% of plaques had VSMCs of a single reporter colour (*Figure 3C*) at both 6.5 and 11 weeks. Where several Confetti colours were detected, cells were typically arranged in contiguous regions. The bias towards lesions containing 1 or 2 confetti colours contrasts with what would be expected by chance if several VSMCs migrated into the lesions. This is consistent with previous reports^7^ and is similar to what we and others found at later stages of plaque development.^6–9^ These findings indicate that activation of VSMC proliferation is a rare event in plaque development, similar to what we observed after injury. We further confirmed restriction to one or two Confetti colours at early plaque stages by stratification according to lesion size, total number of lineage-labelled plaque VSMCs, and vascular bed (*Figure3D* and *E* and Supplementary material online, *Figure S2C*). In particular, the bias for contribution from a single colour is stronger in small compared with large lesions (*Figure 3D*) and in plaques containing few compared with many VSMCs (*Figure 3E*). This is opposite to what would be expected if the oligoclonal nature of VSMCs in mature lesions was due to competition between clones from several VSMCs. We speculate that the slightly increased frequency of contribution by all four Confetti colours in larger lesions (*Figure3D* and *E*) could arise from plaque merging or continuous sporadic VSMC activation during atherogenesis.

The medial layer generally remained mosaic with respect to Confetti-reporter expression. However, in 60% of VSMC-containing lesions, contiguous patches of a single Confetti colour were seen in the media underlying the lesion (see Supplementary material online, *Figure S2D*) and a VSMC clone inside the lesion which expressed the same Confetti reporter as the medial patch was observed in 45/50 cases. This suggests that activation of VSMC proliferation may have occurred prior to migration into the lesion, similar to what we observed after vascular injury. Medial VSMC patches were accompanied by evidence of cell death (in 53 of 53 lesions displaying medial patches), similar to the acellularity observed within remodelled areas in injured arteries (see Supplementary material online, *Figure S1F*). Taken together, this analysis indicates that VSMC investment and proliferation are also low frequency events in atherogenesis.

### 3.3 Induction of VSMC proliferation also occurs at low frequency *in vitro*

To investigate whether the induction of rare VSMC clones observed *in vivo* is dependent on infiltrating immune cells, we analysed proliferation of aortic explants from Myh11-Confetti animals (see Supplementary material online, *Figure S3*). Tissue explants generally displayed persistence of mosaic VSMC labelling after culture, but developed monochromatic patches similar to those observed *in vivo* along explant edges and at forceps pinch-induced regions with VSMC death (see Supplementary material online, *Figure S3B*). Quantification of surfaces expressing the same Confetti protein confirmed that the vast majority remained comparable in size to those detected in freshly isolated vessels (see Supplementary material online, *Figure S3C–E*). On average, 30.5 (±10.8, S.E.M.) large VSMC surfaces were observed in 1 mm^2^ tissue explants after 8 days of culture, whereas these were rare at Day 0 (2.2 ± 1.3, Supplementary material online, *Figure S3E*). Confirming the notion that these resulted from clonal expansion of a small number of cells, similar large VSMC patches were observed in tissue explants from animals with reduced labelling density (see Supplementary material online, *Figure S3F* and *G*).

To test whether proliferation is also restricted to a small number of cells after enzymatic VSMC dissociation, we cultured freshly isolated, lineage-labelled VSMCs from Myh11-Confetti animals mixed with wild-type VSMCs to maintain cell–cell contacts required for VSMC survival and performed live cell imaging periodically. Most Confetti+ cells in these cultures remained as ‘singlets’ surrounded by wild-type cells but small patches of lineage-labelled VSMCs of one colour formed at a frequency of 1.1 (±0.1 S.E.M.) per 1250 Confetti+ VSMCs seeded (see Supplementary material online, *Figure S4*). The clonal expansion of a small fraction of VSMC-lineage-labelled cells *in vitro* indicates that activation of VSMC proliferation is a rare, cell-autonomous event.

### 3.4 Injury induces population-wide, progressive changes in VSMC expression profiles

**Figure 4:**
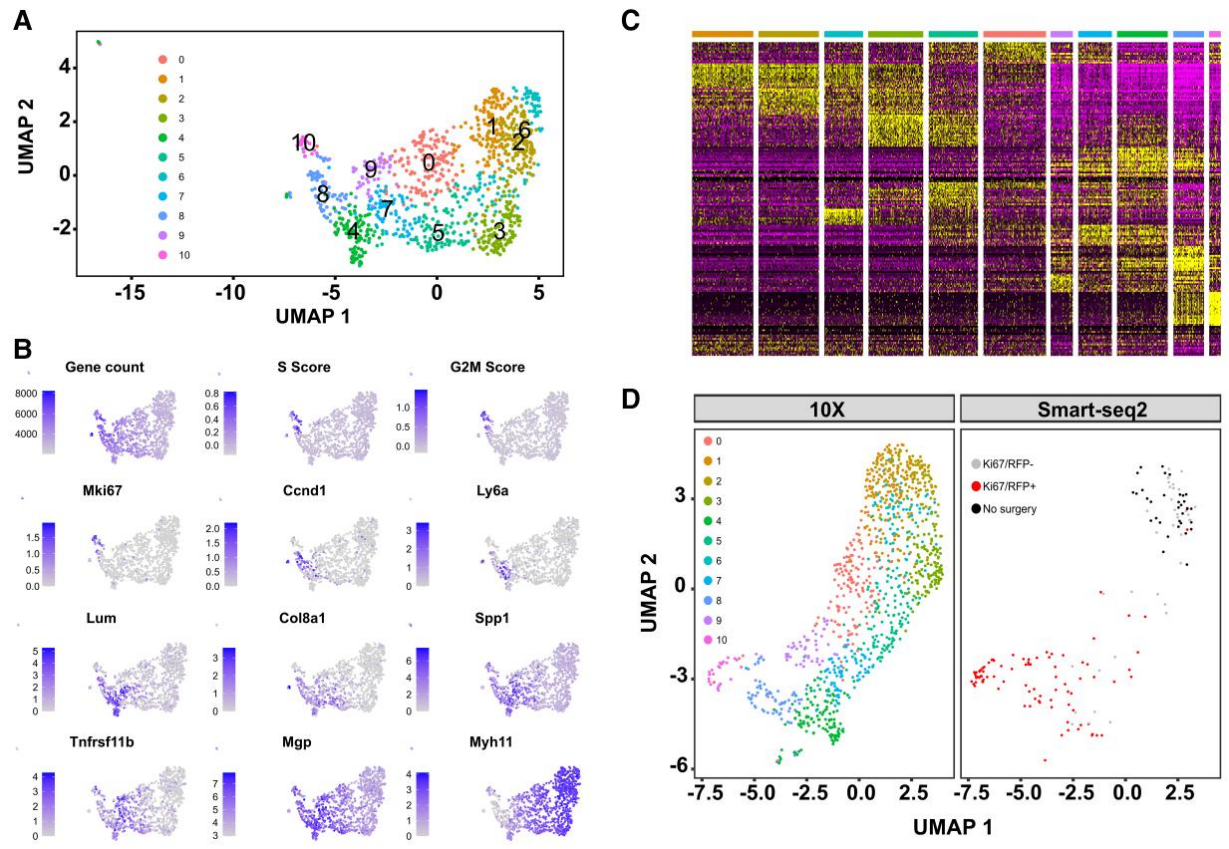
Gradual changes from the contractile to a proliferation-associated expression signature. (*A*) Uniform Manifold Approximation and Projection (UMAP) with cell cluster map for scRNA-seq analysis of 1103 VSMC-lineage label positive cells from left carotid arteries 7 days after ligation. A cell cluster representing a minor VSMC population (Cluster 11, 23 cells) is not shown, see Supplementary material online, *Figure S5*. (*B*) UMAP plots showing gene count, cell cycle scores, and marker gene expression levels using grey-blue scales. (*C*) Heatmap showing expression of the top cluster markers using a scale from purple (low) to yellow (high). Top bar shows cluster affiliation using colour scale from (*A*). (*D*) UMAP after integration of 10× and Smart-seq2 scRNA-seq data (both Day 7), split by dataset. Colours show cell clusters (10 × dataset, left) and index-sort identity (Smart-seq2 dataset, right; cells from unligated control animals in black, and cells from ligated left carotid arteries 7 days after surgery in red (KI67/RFP+) or grey (KI67/RFP-)).

To assess whether the observed low VSMC proliferation frequency reflected a heterogeneous response of distinct VSMC sub-populations, we generated scRNA-seq profiles of lineage-labelled cells 1 week after surgery, at the peak VSMC proliferation frequency (Day 7, D7, *Figure 2J*). We enriched for proliferating VSMCs by cell sorting, using Myh11-EYFP-Ki67/RFP animals. After QC to remove cells with high percentage mitochondrial reads and low numbers of detected genes, the profiles of 1126 cells were assessed for expression of markers of VSMC phenotypic state^38^ and proliferation-associated genes. Most cells formed a single contiguous population that showed gradual variations in marker gene expression (*Figure4A* and *B*). Clustering revealed a separate ‘minor VSMCs’ population, that expressed contractile genes and no-to-low levels of proliferation markers and was removed for display purposes (see Supplementary material online, *Figure S5*), but otherwise cluster borders were not clearly defined. Contractile gene expression (*Myh11*) was high in Clusters 1, 2, 3, and 6, ‘synthetic’ genes (*Mgp*, *Spp1*, *Col8a1*) peaked in Clusters 4, 5, 7, and 9 and cell cycle genes (*e.g. Mki67*, *Ccnd1*) were expressed in Clusters 8 and 10 (*Figure 4B*). Genes induced in VSMC-derived plaque cells (*Lum*, *Tnfrs11b*)^39^ showed overlapping expression with synthetic genes. The expression domain of *Ly6a*, which encodes SCA1 and is expressed in rare VSMCs prior to injury,^13^ partially overlapped that of *Ccnd1* and *Mki67*. We did not observe a distinct cluster of expanded progenitor cells, nor evidence of cluster-specific induction of VSMC-regulators *Klf4* and *Oct4*^5,40^ (see Supplementary material online, *Figure S5C*). Instead, the top cluster markers showed overlapping gene expression gradients across the cell population, similar to the genes characterizing VSMC phenotypes (*Figure 4C* and Supplementary material online, *Table S4*), suggesting that VSMCs after injury adopt states along a continuous axis of phenotypic switching.

Integration of this dataset with Smart-seq2 profiles of index-sorted cells from the same time point showed that *Myh11*-high clusters are similar to VSMCs from healthy vessels (black dots in *Figure 4D*). In contrast, EYFP+ VSMCs expressing the KI67-RFP reporter (red dots in *Figure 4D*) mapped with the *Ccnd1*/*Mki67*-expressing clusters characterized by high S and G2/M scores (*Figure4A* and *B*), verifying that proliferating cells overlap with the most pronounced cell state changes. In addition to rising levels of classical markers of a synthetic VSMC state, the total number of genes detected also increased along this axis (*Figure 4B*). Taken together, these data suggest gradually increasing cell activation along a trajectory from a quiescent-contractile to a proliferative state and that VSMC proliferation is overlapping with cells showing evidence of pronounced, classical VSMC phenotypic switching.

### 3.5 VSMC proliferation-associated injury-response

**Figure 5:**
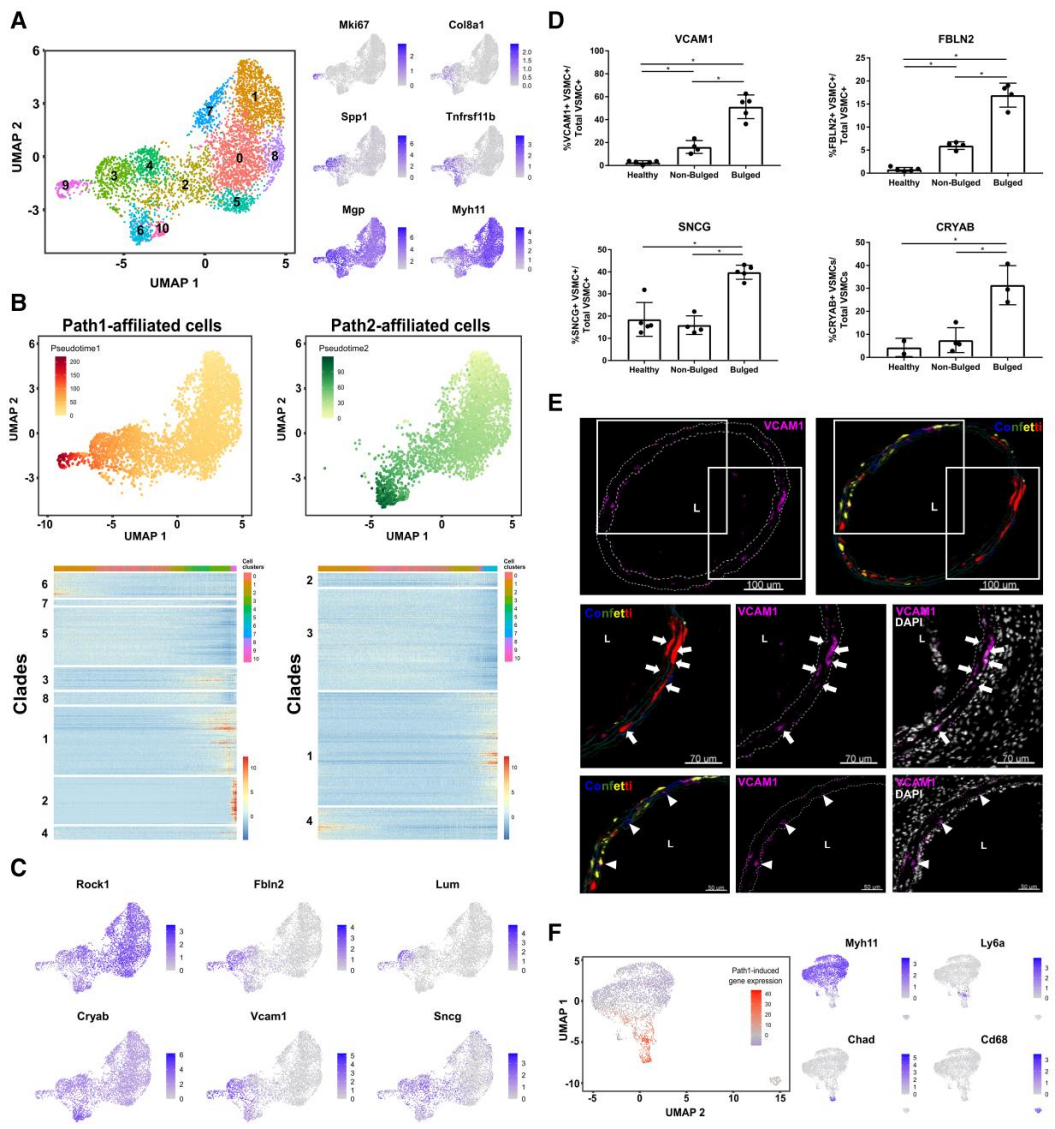
Characterization of injury-induced gene expression at the onset of VSMC proliferation. (*A*) UMAP showing cell clusters (left) and marker gene expression levels using grey-blue scales (right) in a scRNA-seq (10 × Chromium) dataset of 4469 VSMC-lineage label-positive cells from ligated left carotid arteries 5 days after surgery. (*B*) Top panels are UMAPs showing only cells that are part of Path1 (left) or Path2 (right) with pseudotimes for each path indicated using a yellow-red (Path1, left) or yellow-green scale (Path2, right). Lower panels show heatmaps of genes with significant path-associated expression [*P*-adj < 0.05, log(fold-change) > 0.5], clustered into 8 (Path1, left) or 4 (Path2, right) gene clades. (*C*) UMAP showing expression of selected trajectory-associated genes using grey-blue scales. (*D*) Percentage of lineage-labelled VSMCs (VSMC+) expressing FBLN2, CRYAB, SNCG, and VCAM1 in arteries from control animals (healthy), and in non-bulged and bulged regions of injured arteries 5–7 days post-ligation (Myh11-Confetti or Myh11-EYFP animals). Mean and standard deviation are shown. Dots show average of multiple sections from each animal. Asterisk indicates *P* < 0.05 (Welch’s one-way ANOVA and Dunnett’s post-hoc test). (*E*) Representative immunofluorescence staining for VCAM1 in cryosections from bulged regions of ligated carotid arteries from VSMC-lineage-labelled Myh11-Confetti animals 5–7 days after ligation surgery (*n* = 5). Panels show the entire artery (top) and magnified views of boxed regions (middle and lower). Signals for VCAM1 (magenta), Confetti (CFP: blue, RFP: red, YFP: yellow, GFP: green) and DAPI (white) are shown as indicated. Arrows point to VCAM1+ cells in patches and arrowheads point to VCAM1+ singlet VSMCs. The lumen (L) is indicated. Scale bars = 100 µm (top), 70 µm (middle), and 50 µm (lower panels). (*F*) UMAP of scRNA-seq dataset from VSMC-lineage-labelled cells from Myh11-Confetti-Apoe animals fed a high fat diet.^13^ Left, summarized expression of Path1-induced genes (Path1 Clades 1, 2, 3, 4, 8) on a blue (low)-to-red (high) scale. Right, expression of VSMC-derived cell markers (*Myh11*: contractile, *Ly6a*: mesenchymal stem cell-like; *Chad*: osteochondrogenic; *Cd68*: macrophage) in grey-blue scales.

To investigate this idea further, we profiled lineage-labelled (EYFP+) cells at the onset of VSMC proliferation, 5 days after carotid ligation (D5, *Figure 2J*). Similar to the post-surgery Day 7 (D7) cells, D5 VSMCs formed a continuous population displaying anticorrelated, gradual changes in contractile and synthetic markers, and cells expressing *Mki67* and contractile genes clustered at opposite ends of the spectrum (*Figure 5A*). All VSMCs were clearly distinct from adventitial, endothelial, and immune cells, as demonstrated by scRNA-seq analysis of VSMCs together with other arterial cells (see Supplementary material online, *Figure S6*).

Trajectory inference of VSMC-lineage+ cells indicated the existence of two distinct VSMC injury-response pathways, which was confirmed by PAGA analysis (see Supplementary material online, *Figure S7*). The pseudotime axes defining these two paths share a common origin but only Path1 included proliferating *Mki67*+ cells in Cluster 9 (*Figure 5B*). Genes showing significant changes [*P*-adj < 0.05, log(fold-change) > 0.5] along each pseudotime were clustered into gene clades based on correlated gene expression patterns along Path1 or Path2 (*Figure 5B*, lower panels and Supplementary material online, *Table S5*). Contractile genes (e.g. *Myh11* and *Acta2*) and actin cytoskeletal organizers (e.g. *Rock1* and *Lmod1*) showed reduced expression along both pseudotime axes (*Figure5B* and *C* and Supplementary material online, *Table S5*), although downregulation of the contractile programme was less pronounced at D5 compared with D7. Genes with increased expression along the two trajectories showed substantial overlap as well as some significant differences (*Figure 5C* and Supplementary material online, *Table S5*). GO terms associated with functions of modulated VSMCs, including collagen biosynthesis and fibril formation (e.g. *Col1a2*, *Col5a1*, and *Errfi1*), extracellular matrix organization (e.g. *Fbln2*, *Fn1*, *Lum*), response to wounding (e.g. *Fgf2*, *Pdgfa*), and substrate adhesion (e.g. *Cdh13*, *Vcam1*) were enriched in both Path1- and Path2-induced genes (see Supplementary material online, *Figure S8* and *Table S6*). *Cryab* and other heat-shock genes were upregulated along both Path1 and Path2 (*Figure 5C* and Supplementary material online, *Table S5*). Only Path2 showed enrichment for genes associated with protein refolding. Interestingly, misfolded proteins have been linked to a VSMC cholesterol response^41^ and suggested to be anticorrelated with Myocardin-related factors.^42^ In contrast, genes associated with cell cycle regulation, such as *Cdk1*, *Mki67*, *Ccnd1* were specifically induced along Path1 (see Supplementary material online, *Table S7*), consistent with the selectivity in activation of VSMC proliferation observed above. *Bona fide* cell cycle genes, such as *Top2a*, were restricted to *Mki67*+ cells (Cluster 9) and mapped to Path1 Clade 2 (see Supplementary material online, *Table S5*). However, the expression domains of other genes in Path1-induced gene clades (Clades 1, 3, 4, 8) were broader (e.g. *Sncg*) and many spanned both cells in Cluster 9 and the pre-proliferative cell cluster (Cluster 3) (e.g. *Fbln2*, *Vcam1*, *Lum*, *Figure5B* and *C*). To determine the tissue location of cells with injury-induced expression profiles, we performed immunostaining in cross-sections from lineage-traced animals (*Figure5D* and *E* and Supplementary material online, *Figures S9–12*). Consistent with the scRNA-seq findings, expression of VCAM1, FBLN2, and SNCG was more abundant in VSMCs in D5–7 post-injury compared with healthy control arteries (*Figure 5D*). FBLN2 and VCAM1 were expressed in very few VSMCs prior to injury, whereas SNCG was expressed in 20% of VSMCs in control arteries, reflecting the transcript levels (*Figure 5C–E* and Supplementary material online, *Figures S9–S11*). In injured arteries, expression of all markers tested was increased in ‘bulges’, which are the predominant site of injury-induced VSMC proliferation, compared with non-bulged arterial regions (*Figure 5D*). Alpha-crystallin B chain (CRYAB) protein expression showed a similar induction pattern (see Supplementary material online, *Figure S12*), confirming that increased detection of the heat-shock gene *Cryab* by scRNA-seq is not dissociation associated.^43^ Path1-induced VCAM1 expression was detected in the majority of VSMCs within clonal patches (87 ± 5%), compared with 51 ± 14% of total VSMCs in bulged regions (*Figure 5E*). VCAM1 expression was detected in singlet VSMCs in both bulged and non-bulged regions (*Figure5D* and *E* and Supplementary material online, *Figure S9*), however, the two-fold enrichment in patches, confirms that the computationally inferred Path1 trajectory is associated with increased VSMC proliferation.

Analysis of Path1-induced genes in VSMC-derived plaque cells^13^ suggested that the injury-induced trajectory towards VSMC proliferation is relevant for atherogenesis. Genes showing increased expression along the proliferation-associated Path1, include *Lum* and *Tnfrsf11b* that were detected in modulated VSMCs by scRNA-seq analysis in vascular disease models.^39,44–47^ Furthermore, as shown in *Figure 5F*, expression of Path1 injury-response genes overlapped the expression domains of markers of phenotypically modulated, plaque-resident VSMCs^13^ (*Chad*, *Ly6a*). Immunostaining confirmed that FBLN2, which is induced along Path1, is expressed in VSMC-derived cells within the core of atherosclerotic lesions in Myh11-Confetti-Apoe animals and was also detected in αSMA-stained cells in human carotid artery plaques (see Supplementary material online, *Figure S13*), indicating that the injury-induced Path1 signature is also activated by VSMCs in human disease.

This analysis identifies two related but distinct injury-responses in VSMCs that are predominantly induced in arterial regions undergoing remodelling-associated changes. Genes induced as VSMCs transition towards VSMC proliferation are also expressed in atherosclerosis models and human lesions, suggesting that the VSMC injury-response is disease relevant.

### 3.6 VSMCs in healthy arteries with partial transition towards VSMC proliferation

**Figure 6:**
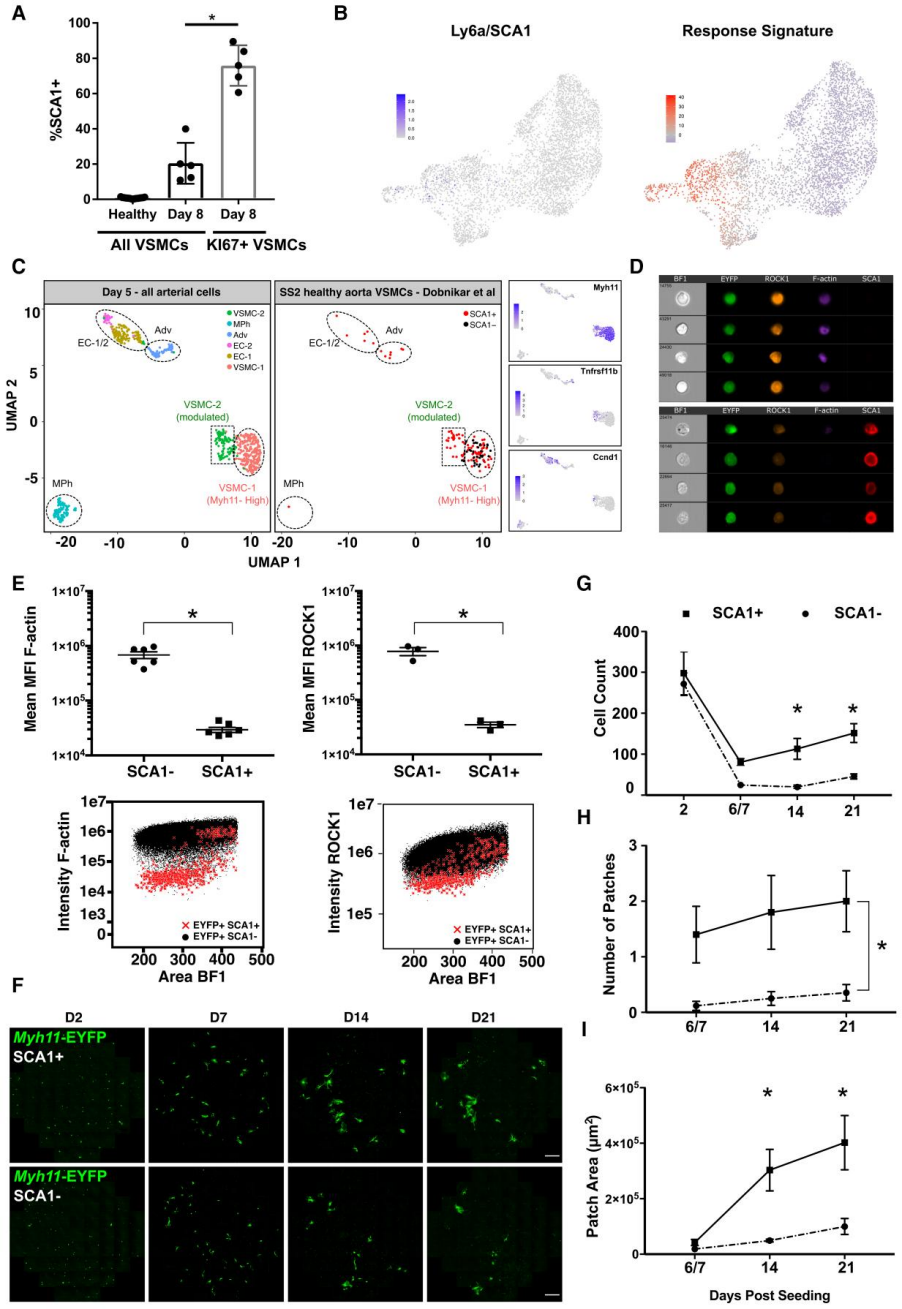
SCA1 marks VSMCs with increased proliferative capacity. (*A*) Percentage of SCA1-expressing cells out of all EYFP+ VSMCs (black bars) or EYFP+KI67/RFP+VSMCs (grey bar) in left carotid arteries of Myh11-EYFP-KI67/RFP animals analysed by flow cytometry, showing data for individual animals (dots), mean, and standard deviation [*n* = 17 (healthy) or 5 (Day 8 post-ligation)]. (*B*) UMAP of D5 VSMC scRNA-seq dataset showing expression of Ly6a/SCA1 using a scale from grey-blue (top) or summarized expression of the ‘Response Signature’ identified in SCA1+VSMCs from healthy arteries^13^ on a blue-red scale (lower panel). (*C*) Integration of scRNA-seq profiles of all arterial cells analysed 5 days post-carotid ligation (Day 5 all) with Smart-Seq2 dataset of cells from healthy arteries.^13^ Left panel shows cell cluster identity in the ‘Day 5 all’ dataset (see Supplementary material online, *Figure S6*), middle panel shows SCA1+ (red) and SCA1− (black) VSMCs from healthy arteries and right-hand panels show expression of *Myh11*, *Tnfrsf11b*, and *Ccnd1* in the integrated dataset. (*D*) ImageStream bright field (BF) and fluorescence images of medial cells from the aortae of VSMC-lineage-labelled Myh11-EYFP animals stained for SCA1 (red), ROCK1 (orange), and F-actin (magenta, phalloidin) with VSMC-lineage label (EYFP) in green. (*E*) Quantification of (*D*). Dots show mean of MFI (mean fluorescence intensity) for SCA1− and SCA1+ cells (gating, see Supplementary material online, *Figure S14*) from each animal (F-actin, *n* = 6; ROCK1, *n* = 3), and mean and S.E.M. across animals are indicated. Asterisks: *P* < 0.05, *t*-test. (*F–I*) Comparison of clonal proliferation for SCA1+ or SCA1− VSMCs (EYFP+) from Myh11-EYFP animals mixed with medial cells from wild-type animals in a 1:9 ratio. (*F*) Representative live confocal images. EYFP signals are shown in green. Scale bar = 100 μm. Quantification of cell count per well (*G*), number of patches per well (*H*), and mean area of patches per well (*I*) for wells containing EYFP+SCA1+ (squares, solid lines) and EYFP+SCA1− cells (circles, dotted lines) at indicated time points post-seeding. Plots show mean and S.E.M. of cells from three animals analysed separately. Statistical significance (asterisks indicate *P* < 0.05) of cell count differences at individual days was tested by ANOVA (*G*); patch numbers were fitted to a generalized linear model (*H*); multiple linear regression was performed on log-transformed data for patch area (*I*).

We previously identified a small number of VSMCs in healthy arteries that are marked by SCA1 and show heterogeneous expression of a ‘Response Signature’ suggestive of cell activation.^13^ Here, we show that the frequency of SCA1+ VSMCs increases after injury with kinetics similar to that of KI67-induction and that proliferating, KI67+ VSMCs are enriched for SCA1-expression (*Figure 6A* and Supplementary material online, *Figure S14A*). Interestingly, the ‘Response Signature’ expressed by SCA1-positive VSMCs in healthy arteries^13^ was also induced in Path1-specific cells (*Figure 6B*). These results indicate that SCA1 expression is linked with induction of VSMC proliferation and that SCA1+ VSMCs in healthy vessels marks a state similar to that induced by injury.

To directly compare SCA1+ VSMCs from healthy animals with the injury-response states, we used previously generated Smart-seq2 profiles^13^ for integration with scRNA-seq data of arterial cells generated 5 days after surgery, where a Myh11-low, modulated VSMC cluster was detected (VSMC-2, Supplementary material online, *Figure S6*). All SCA1-negative VSMCs from healthy arteries clustered with *Myh11*-high VSMCs (VSMC-1, *Figure 6C* and Supplementary material online, *Figure S15*). In contrast, 25/90 SCA1+ VSMCs isolated from healthy animals mapped to phenotypically modulated VSMCs expressing *Tnfrsf11b* and *Ccnd1* (VSMC-2).^13^ This analysis confirms the previously observed heterogeneity of SCA1+ VSMCs from healthy arteries^13^ and indicates that a subset of these SCA1+ cells corresponds to a transitional state towards VSMC proliferation. Further supporting this idea, SCA1-expressing VSMCs had significantly lower levels of Rho-associated coiled-coil kinase (ROCK1) compared with their SCA1-counterparts (*Figure6D* and *E*), consistent with reduced *Rock1* transcripts along Path1 during the injury-response (*Figure 5C*). SCA1-marked cells from healthy arteries also displayed altered cytoskeleton, illustrated by reduced phalloidin staining, compared with their SCA1-negative counterparts (*Figure 6D and E* and Supplementary material online, *Figure S14B* and *C*).

We then adapted the clonal proliferation assay to test for a link with cell proliferation. SCA1+ or SCA1− cells from aortas of lineage-labelled healthy Myh11-EYFP animals were mixed with wild-type medial VSMCs and EYFP imaged periodically over 3 weeks (*Figure 6F*). There was no difference in cell count 2 days after seeding, demonstrating equal survival of SCA1+ and SCA1− cells. However, after 1 week of culture, more lineage-labelled cells were detected in SCA1+ compared with SCA1− samples and a significant difference persisted throughout the experiment (*Figure6F* and *G*). The increasing cell number resulted from emergence of patches of EYFP+ cells, indicating clonal proliferation (*Figure 6F and H*). In SCA1+ samples, 1–3 patches were observed per well 1 week after seeding (2.4 patches/well predicted by Poisson regression analysis). Patch number remained approximately constant, whereas the size of individual patches increased over time for SCA1+ samples (*Figure6H* and *I*). In contrast, wells containing SCA1− EYFP+ cells did not contain patches after 1 week of culture and at later time points patches were observed at lower frequency in SCA1− compared with SCA1+ cultures (4/18 wells, *Figure 6H*).

This analysis demonstrates that SCA1-expressing cells in healthy vessels are phenotypically and functionally distinct from the bulk of VSMCs. The eight-fold increased patch number and faster kinetics of clone formation for SCA1+ VSMCs show that some SCA1+ cells have a proliferative advantage, but that proliferation is not restricted to SCA1-expressing VSMCs from healthy vessels.

**Figure 7:**
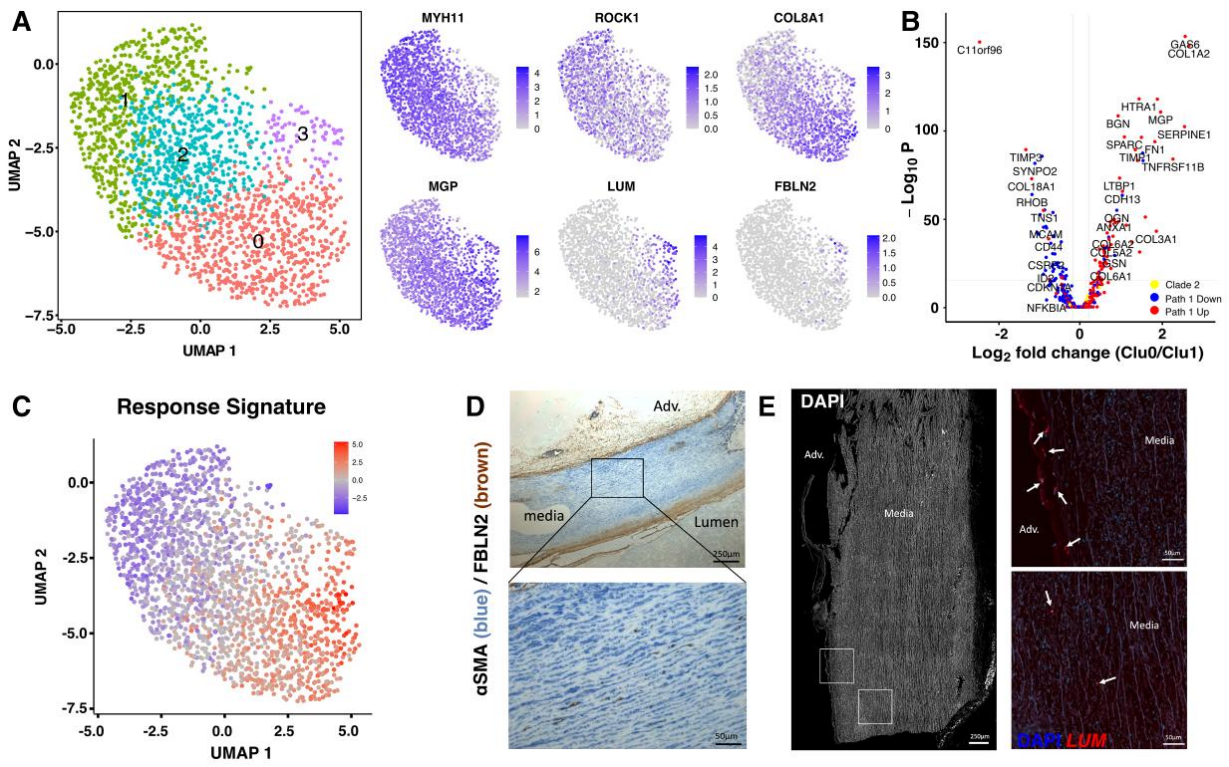
VSMC heterogeneity in human arteries. (*A*) UMAP of 1978 medial cells from a healthy human aorta showing cluster map (left) and expression level of marker genes using grey-blue scales (right). (*B*) Volcano plot showing differential expression between Cluster 0 and Cluster 1 cells. Dots represent genes showing Path1-induced (red, Path1 Clades 1, 3, 4, 8), Path1-down-regulated (blue, Path1 Clades 5, 6, 7) or cell cycle-associated (yellow, Path1 Clade 2) expression in the mouse D5 dataset. (*C*) Summarized expression of the ‘Response Signature’ identified in SCA1+ VSMCs from healthy mouse arteries,^13^ shown on the UMAP of human medial cells in a blue-red scale. (*D*) Immunohistochemistry co-staining for FBLN2 (brown) and αSMA (blue) in section of healthy human aorta. Representative of six individuals. Scale bar = 250 μm (top), 50 μm (lower panel). (*E*) RNA scope *in situ* hybridization of lumican in section of healthy human aorta. Left panel shows DAPI signal in white, and boxed regions are magnified in right panels with signals for *LUM* mRNA (red) and DAPI (blue). Arrows point to *LUM*+ cells in the media or adventitia (lower left). Representative of five individuals. Scale bar = 250 μm (left), 50 μm (right).

SCA1 does not have an obvious human orthologue, preventing direct translation to human disease. We therefore performed scRNA-seq of medial cells isolated from healthy human aorta. As expected, a single population of cells expressing contractile VSMC markers was detected. However, reduced levels of contractile genes were observed in Cluster 3 and a subset of cells in Cluster 0. Anticorrelating with *MYH11*, gradually higher expression of *COL8A1*, *MGP*, and other synthetic genes was detected through Clusters 2, 0, and 3, suggesting that cells with different extents of phenotypic switching exist in human vessels (*Figure 7A*). Interestingly, genes that are downregulated along Path1 in the murine injury model showed a lower expression in Cluster 0 vs. Cluster 1. In contrast, injury-induced Path1-associated genes were higher in Cluster 0 (*Figure 7B*). Additionally, the ‘Response Signature’ identified in murine SCA1+ VSMCs was also differentially expressed across the human VSMC population with a maximum level in Cluster 1, further suggesting that human VSMCs show heterogeneity akin to what we found in mouse (*Figure 7C*).

We then analysed healthy human aorta sections for localization of FBLN2 and LUM, which both show reciprocal expression compared with contractile genes in mouse (*Figure 5C*) and human VSMCs (*Figure 7A*). FBLN2 was detected in αSMA+ cells in the medial layer (*Figure 7D*), but at lower frequency compared with lesions (see Supplementary material online, *Figure S13B*). *In situ* hybridization also confirmed sporadic, rare expression of *LUM* in cells of the medial layer of healthy arteries, in addition to more frequent and high-level expression in adventitial fibroblasts (*Figure 7E*). Both LUM+ and FBLN2+ cells were dispersed in the medial layer of human arteries and did not cluster to specific regions that could represent early-stage vascular disease. Taken together, these results suggest that healthy human arteries also harbour VSMCs displaying the hallmarks of disease-associated phenotypic transition identified in mouse models.

## 4. Discussion

We here demonstrate that oligoclonal VSMC contribution to vascular lesions results from activation of proliferation in a small subset of pre-existing VSMCs based on (i) the gradually increasing number of VSMC clones over time after ligation surgery, (ii) a low frequency of VSMC-lineage+ KI67-RFP-reporter expressing cells, (iii) the high frequency of investment of VSMCs of a single colour at early stages of plaque development, and (iv) rare induction of clonal proliferation *in vitro*. We show that the medial vascular layer contains a substantial proportion of CD45+ cells at the onset of VSMC proliferation, which is consistent with previous reports.^37^

The gradually changing cell states observed by scRNA-seq after injury are at odds with what would be expected if proliferation arises from a specific progenitor population. Rather, the scRNA-seq analysis suggests population-wide responses to injury with cell-to-cell variations in the extent of phenotypic modulation. Pre-proliferative cells along the proliferation-associated Path1 share an expression profile with the atypical SCA1+ VSMCs we previously identified in healthy arteries,^13^ suggesting that these represent cells that have partially transitioned towards proliferation. While SCA1 may not be functionally important, we propose that SCA1 in mice marks VSMCs that are primed to respond to inductive signals such as inflammation or injury. In accordance with this idea, SCA1+ VSMCs show increased capacity for clonal expansion *in vitro*, whereas SCA1− cells proliferated less and with slower kinetics. We speculate that formation of patches in SCA1− sorted samples results from *in vitro* induction of a SCA1 signature as previously documented in cultured VSMCs.^13^ This is consistent with a suggested increased proliferation and plasticity of SCA1+ cells in atherosclerotic lesions^46,47^ where SCA1+ VSMC-lineage+ plaque cells have been proposed to generate diverse VSMC-derived cell states identified by scRNA-sequencing.^13,39,44,46,47^

Our finding that SCA1+ cells in healthy arteries are similar to cells found in disease aligns with the observation that modulated VSMCs mapping with SCA1+ plaque cells were identified in Apoe−/− animals prior to HFD-induced atherosclerosis.^39^ The suggestion that SCA1-expressing VSMCs in healthy vessels are involved in VSMC proliferation and disease development is challenged by results of recent SCA1-lineage tracing, which did not find contribution of SCA1-lineage-labelled cells in atherosclerosis and vascular injury models.^48,49^ We note that *Ly6a*-transcript levels are lower in VSMCs compared with ECs and adventitial cells (see Supplementary material online, *Figure S6B*), suggesting that lack of medial cell labelling in the SCA1-CreERt2 model^48^ could be because the CRE activity is below the threshold needed for recombination in VSMCs. Notably, efficient recombination in the Myh11-CreERt2 model requires a series of ten tamoxifen injections.^4^Alternatively, proliferation could be induced in very few pre-existing VSMCs in a stochastic manner, regardless of initial expression state, or result from negative feedback mechanisms induced by proliferating cells to prevent neighbouring cells from exiting quiescence, akin to lateral inhibition. Experimental testing of VSMC priming using alternative drivers for lineage labelling is not trivial, as genes induced in modulated VSMCs, such as *Fbln2* and *Lum*, are also expressed by other cell types in the vasculature, necessitating a dual lineage labelling approach.^44,50^

The transcriptional signature defined by the contractile-to-proliferative axis in post-injury VSMCs shares considerable overlap with transcriptional states of VSMC-derived cells in other vascular disease models, including atherosclerosis and aneurysm formation.^12,39,44–47^ We therefore propose that the mechanisms acting at the onset of VSMC proliferation after injury may also regulate early steps of disease development. Consistently, we observe clonal VSMC contribution in very small atherosclerotic plaques even before formation of the fibrous cap, which appears to be in contrast to a study suggesting that plaque VSMC investment results from migration along the fibrous cap.^7^ Despite these apparent discrepancies, our findings are in accordance with the idea that a phenotypically modulated, plastic cell state underpins atherosclerotic lesion VSMC infiltration.^13,44,46,51^ Such a state has been defined by expression of *Lgals3*, which is present prior to cap formation,^44^ or Ly6a/SCA1,^39,46,47^ consistent with the observation of proliferation-associated SCA1+ cells in our dataset. We did not detect differential expression of Notch pathway genes at these time points after carotid ligation. Notch3 was recently suggested to regulate clonal proliferation of smooth muscle cells in pulmonary arteries,^52^ whereas Notch1 (but not Notch3) mutants affected neointima formation in carotid arteries.^53^ VSMC proliferation was associated with cell death in both arterial injury and in the atherosclerosis model, similar to what has been suggested.^54,55^ It is therefore tempting to speculate that cells forming Path2 in the scRNA-seq analysis constitute dying cells. However, we did not find evidence of enrichment for apoptosis or cellular senescence in genes showing induction along Pseudotime 2, compared with Pseudotime 1 (see Supplementary material online, *Tables S5* and *S6*).

We propose that the previously reported high medial replication index (>20%) 5 days after carotid ligation surgery^15^ is at least partially due to proliferation of non-VSMCs, likely infiltrating immune cells. Alternative causes of high medial BrdU incorporation^15^ include DNA damage repair or S-phase entry of VSMCs that either die or remain blocked in G2/M. The model system used here precludes longitudinal monitoring of VSMC clonality *in vivo* and we cannot rule out that the VSMC patches observed at late time points are derived from other cells than those observed at early time points. However, VSMC doublets were not frequently detected in low-labelling experiments *in vivo*^6^ or in culture (see Supplementary material online, *Figures S3* and *S4*), which does not support a more widespread induction of VSMC proliferation that is not sustained. Furthermore, we did not observe increased expression of cell cycle regulators that could indicate a G2/M block along the alternative injury-response (Path 2), that was not associated with proliferation.

The observation that only a subset of medial VSMC clones contribute to neointima formation following injury could be due to differences in migratory capacity between clones, or migration to the intimal space might be restricted to clones juxtaposed with fenestrations^56^ or breaks in the internal elastic lamina. The identification of distinct phases of VSMC activation adds additional layers to the knowledge of VSMC regulation. It will therefore be important to understand whether documented VSMC regulators^1,5,57^ and novel pathways—such as retinoic acid signalling and efferocytosis identified by scRNA-seq analysis of atherosclerotic plaque cells^46,47^—impact specifically on VSMC priming, induction of proliferation, and/or VSMC migration into the vessel intima.

The activation of VSMC proliferation in a small number of cells has implications for the development of effective treatment strategies in cardiovascular medicine. We propose that manipulation of genes and pathways identified here would impact VSMC expansion, suggesting that the signatures associated with VSMC heterogeneity will enable future analyses of VSMC priming dynamics and the interaction with genetic predisposition to cardiovascular disease.

## Supplementary material

Supplementary material is available at *Cardiovascular Research Europace* online.

## Authors’ contributions

M.D.W. led the clonal dynamics studies in animal models. J.L. led the *in vitro* studies and analysed human samples. S.O. led the computational analysis of murine datasets. J.C.K.T, A.L.T., L.D., J.C., J.L.H., N.L.F., A.F., K.F., A.K.U., M.R.B. contributed to generation, analysis, and interpretation of experiments. M.S. and H.F.J. conceived the study. M.D.W., J.L., S.O., and H.F.J. wrote the manuscript with input from all authors.

## Supplementary Material

cvac138_Supplementary_Data

## Data Availability

The scRNA-seq datasets generated in this study have been deposited to the Gene Expression Omnibus (GEO) repository (GSE162167). The scRNA-seq datasets from VSMC-lineage-labelled plaque cells from HFD-fed Myh11-Confetti-Apoe animals and the Smart-seq2 dataset from healthy Myh11-Confetti animals are available from GEO (GSE117963).^13^

## Notes

Translational perspective The demonstration that vascular smooth muscle cell (VSMC) oligoclonality arise from activation of proliferation in a small number of VSMCs, but that these cells are unlikely to correspond to a dedicated progenitor population, has implications for the development of effective treatment strategies in cardiovascular medicine. We propose that the genes and pathways we identify as associated with activation of VSMC proliferation could be manipulated to impact VSMC expansion in disease.
