# Correction to: Cellular mechanisms of oligoclonal vascular smooth muscle cell expansion in cardiovascular disease

**DOI:** 10.1093/cvr/cvae251

**Published:** 2024-12-16

This is a correction to: Matt D Worssam, Jordi Lambert, Sebnem Oc, James C K Taylor, Annabel L Taylor, Lina Dobnikar, Joel Chappell, Jennifer L Harman, Nichola L Figg, Alison Finigan, Kirsty Foote, Anna K Uryga, Martin R Bennett, Mikhail Spivakov, Helle F Jørgensen, Cellular mechanisms of oligoclonal vascular smooth muscle cell expansion in cardiovascular disease, *Cardiovascular Research*, Volume 119, Issue 5, May 2023, Pages 1279–1294, https://doi.org/10.1093/cvr/cvac138

In the original published version of this article, Figure 6E includes an incorrect graph for the quantification of ROCK1 protein levels. The raw data panel is correct and the corrected quantification panel consistently shows a significantly lower ROCK1 level in SCA1+ compared to SCA1- cells (albeit with smaller effect size).

This error does not affect the statements made in the manuscript nor the conclusions of the study. The Authors wish to correct this to include the correct graph for quantification of ROCK1 protein levels.

**Figure:**
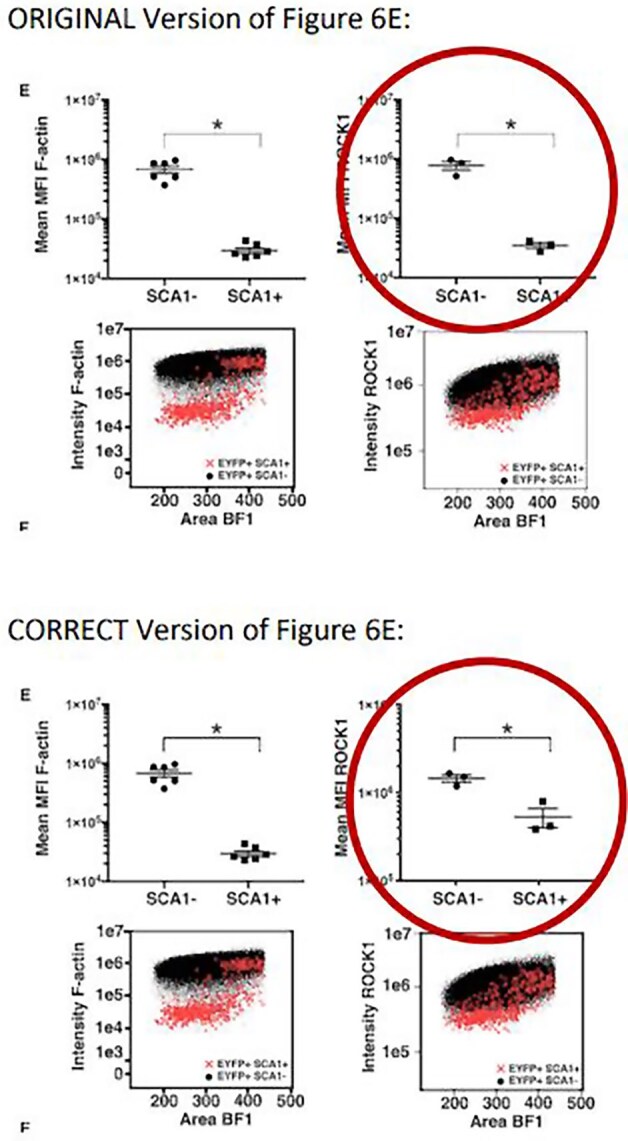


The error is outlined only in this notice to preserve the version of record.

