# Supplementary material for: Cellular mechanisms of oligoclonal vascular smooth muscle cell expansion in cardiovascular disease
Source: Cardiovasc Res. 2022 Aug 22;119(5):1279–94. doi: 10.1093/cvr/cvac138 (PMC10202649; doi:10.1093/cvr/cvac138)
Supplement: cvac138_Supplementary_Data [file cvac138_supplementary_data.zip › MDW_JL_SO_Supplementary.pdf]

SUPPLEMENTARY MATERIAL

Worssam/Lambert/Oc *et al.*

Content of this document

Detailed Methods
Supplementary References
Supplementary Figures I-XV

Separate files

Supplementary Table I: Antibodies used in this study
Supplementary Table II: Settings used for confocal microscopy
Supplementary Table III: Carotid arteries analyzed by whole mount imaging
Supplementary Table IV: Top marker genes for cell clusters in mouse D7 scRNA-seq data
Supplementary Table V: Genes showing significant association with Path1 and Path2 in mouse D5 scRNA-seq data
Supplementary Table VI Gene ontology analysis of genes showing induced expression along Path1 and Path2 in mouse D5 scRNA-seq dataset
Supplementary Table VII: Expression of cell cycle regulators in D5-EYFP scRNA-seq cell clusters

List of Supplementary Figures:

Supplementary Figure I: Examples and quantification of whole-mounted left carotid arteries of VSMC-lineage labeled Myh11-Confetti animals (Related to Figure 1, 2)
Supplementary Figure II: Quantification of plaques in VSMC-lineage labeled Myh11-Confetti-Apoe animals (Related to Figure 3)
Supplementary Figure III: Rare activation of VSMC proliferation in tissue explant cultures
Supplementary Figure IV: Clonal proliferation of dissociated VSMCs in culture
Supplementary Figure V: Feature plots of mouse D7 scRNA-seq dataset including cell cluster 11 (Related to Figure 4)
Supplementary Figure VI: VSMCs form a single population distinct from macrophages, endothelial and adventitial cells 5 days after injury (Related to Figure 5)
Supplementary Figure VII: Trajectory inference for mouse D5 scRNA-seq data (Related to Figure 5)
Supplementary Figure VIII: Gene ontology (GO) analysis of genes showing trajectory-induced expression in mouse D5 scRNA-seq dataset (Related to Figure 5)
Supplementary Figure IX: Immunostaining for VCAM1 in sections from mouse carotid arteries (Related to Figure 5)
Supplementary Figure X: Immunostaining for FBLN2 in sections from mouse carotid arteries (Related to Figure 5)
Supplementary Figure XI: Immunostaining for SNCG in sections from mouse carotid arteries (Related to Figure 5)
Supplementary Figure XII: Immunostaining for CRYAB in sections from mouse carotid arteries (Related to Figure 5)
Supplementary Figure XIII: Immunostaining for FBLN2 in atherosclerotic lesions (Related to Figure 5)
Supplementary Figure XIV: Flow cytometry and ImageStream analysis (Related to Figure 6)
Supplementary Figure XV: SCA1+ VSMCs from healthy arteries are similar to modulated VSMCs following injury (Related to Figure 6)

Detailed Methods

Animals and procedures

Animal experiments were approved by the local ethics committee and were performed according to UK Home Office regulation under project license P452C9545. All alleles have been described previously; Myh11-CreERt2 is a Y-linked transgene that confers expression of a tamoxifen-inducible Cre recombinase in smooth muscle cells[1,2], Rosa26-Confetti[3] and Rosa26-EYFP[4] are Cre-recombination reporter alleles, KI67-RFP is an insertion in the Mki67 locus resulting in expression of a KI67-RFP fusion protein[5] and the mutant Apoe allele sensitizes mice to high fat diet (HFD)-induced atherosclerosis development[6]. Myh11-CreERt2 is Y-linked, so all VSMC lineage-tracing experiments were performed using males.

Unless indicated, VSMC lineage labeling was done by 10 intraperitoneal tamoxifen injections (1 mg in corn oil) over 2 weeks into Myh11-CreERt2, Rosa26-Confetti (Myh11-Confetti), Myh11-CreERt2, Rosa26-Confetti, $Apoe^{-/-}$ (Myh11-Confetti-Apoe), Myh11-CreERt2, Rosa26-EYFP (Myh11-EYFP), Myh11-CreERt2, Rosa26-EYFP, $Apoe^{-/-}$ (Myh11-EYFP-Apoe) and Myh11-CreERt2, Rosa26-EYFP, KI67-RFP (Myh11-EYFP-Ki67/RFP) animals. All animals were rested for at least 1 week to allow tamoxifen washout. The tamoxifen administration protocol does not result in equal induction of the four reporter proteins. We find that GFP induction occurs at lower frequency, similar to previous studies[7].

Carotid ligation was performed as previously described[7]. Briefly, animals were given pre-operative analgesic (~0.1 mg/kg body weight, Buprenorphine) subcutaneously, anaesthetized with isoflurane by inhalation (2.5-3%, 1.5 L/min for induction, maintained at 1.5%) and the left carotid artery was tied off with a silk suture. Animals were culled 2-28 days after surgery by CO2 asphyxiation and perfused with cold phosphate buffered saline (PBS) before tissue removal. Lineage-labeled Myh11-Confetti-Apoe animals were fed a HFD (Special Diets Services, containing 21% fat and 0.2% cholesterol) for 6.5-24 weeks as indicated, starting 1 week after the last tamoxifen injection. Animals were euthanized by cervical dislocation or increasing CO2 concentration.

Tissue processing for imaging

Mouse arteries dissected free of adipose tissue were fixed in fresh 4% formaldehyde (Sigma) for 20 minutes at room temperature, stained with 4', 6-diamidino-2-phenylindole (DAPI, 1 µg/mL in PBS) overnight at 4 °C, cleared using RapiClear 1.52 (Sunjin Lab) for at least 5 hours and mounted in RapiClear 1.52 using iSpacers (Sunjin Lab) for imaging. Fixed arteries from Myh11-Confetti-Apoe, Myh11-EYFP-Apoe animals, and post-imaging Myh11-Confetti carotid arteries, were frozen in optimal cutting temperature (OCT, Tissue-Tek) compound after cryopreservation in 30% (w/v) sucrose in PBS and equilibration in sucrose:OCT (1:1). Sections were cut on a cryostat microtome onto Superfrost™ Ultra (thin, 14 µm) or Superfrost™ Ultra Plus Slides (thick, 100 µm)(Thermo scientific) for immunostaining or directly stained with DAPI (1 µg/mL in PBS) for 20 minutes at room temperature before mounting in RapiClear 1.52.

Human arteries were formaldehyde-fixed and paraffin-embedded (FFPE) and 4 µm sections cut onto Superfrost™ Ultra Plus slides.

Tissue explants
Aortas were isolated from VSMC-lineage labeled animals (Myh11-Confetti or Myh11-EYFP), dissected free of adipose tissue and pre-digested in Opti-MEM (Gibco) supplemented with 1 mg/mL Collagenase IV (Invitrogen) and 1 U/mL Elastase (Worthington) for 10 minutes at 37˚C to allow removal of the adventitial layer. Arteries were kept in Opti-MEM overnight and 1 mm^2 tissue sections cut with minimal mechanical manipulation. Explants were pinched with sharp forceps to create an internal injury site and embedded in Matrigel in an 8-well chamber slide (Ibidi). Growth medium (Opti-MEM supplemented with 10% (v/v) fetal bovine serum (FBS), 100 U/mL penicillin, 100 mg/mL streptomycin and 20 ng/mL PDGF was added and changed every 2-3 days. Explants were fixed directly (day 0) or after 8 days of culture in 2% (w/v) methanol-free paraformaldehyde (Thermo Fisher) and 0.5% (w/v) glutaraldehyde (Sigma Aldrich) in PBS for 30 minutes at 4 °C. After washing in PBS, explants were mounted in RapiClear 1.47 (Sunjin Lab) using iSpacers (Sunjin Lab).

Immunostaining and in situ hybridization of tissue sections
Antibodies used for staining are described in Supplementary Table I. Carotid artery cryosections were thawed, rinsed in PBS and permeabilized for 20 minutes in 0.5% (v/v) Triton X-100 (Sigma Aldrich) in PBS at room temperature. Sections were blocked for 1 hour at room temperature in 1% (w/v) bovine serum albumin and 10% (v/v) of either normal goat serum (Dako), or normal donkey serum (Abcam) in PBS and incubated with primary antibody or isotype controls (rabbit IgG Abcam, ab37415 or rat IgG2b, Biolegend, 400626), diluted in blocking buffer overnight at 4°C or 30 minutes at 37˚C. Following 3x 5 minutes washes in PBS incubation, Alexa-647 conjugated secondary antibodies were added for 1 hour at room temperature, sections were washed 2x 5 minutes in PBS and nuclei stained with DAPI (1 µg/mL, 10 minutes at room temperature) before rinsing in PBS and mounting in RapiClear 1.52 (Sunjin Lab).

FFPE sections were dewaxed in Xylene and rinsed in water before antigen retrieval in Citrate-based Antigen Retrieval Solution (pH6, Vector Labs, 30 minutes in an Aptum 2100 Retriever Pressure Cooker). Samples were blocked first in Peroxidase Blocking Solution (Abcam, 10 minutes) then in antibody diluent (SignalStain, 10 minutes). Blocked sections were incubated with anti-FBLN2 (Abcam, ab251662) or isotype control (Abcam, ab37415) in antibody diluent overnight at 4°C, washed 3x in PBS, stained with HRP-conjugated anti-Rabbit (Cell Signaling Technology, 8114) in antibody diluent for 30 minutes at room temperature, washed 3x in PBS and HRP visualized using DAB peroxidase substrate (SignalStain). After rinsing in water, sections were stained with anti-αSMA (DAKO, M0851) in Superblock diluent (Thermo Fisher) for 1 hour at room temperature, washed 3x in PBS and incubated with biotin-coupled anti-Mouse (DAKO, E0433) in Superblock diluent for 30 minutes at room temperature, washed 3x in PBS and incubated in Vectastain avidin-coupled alkaline phosphatase (AP) reagent (Vector Labs) for 30 minutes, before rinsing in PBS and incubating with Blue AP substrate solution (Vector Labs) for 20 minutes, all at room temperature. Finally, sections were washed in PBS and mounted in VectaMount mounting media (Vector Labs).

RNA in situ hybridisation was performed using RNA Scope® Multiplex Fluorescent v2 kits, according to the manufacturer’s instructions (ACD). All experiments used Hs-LUM-C1 probes coupled to Opal™ 690. Images were taking using a ZEISS Axioscan slide scanner.

Confocal microscopy and image analysis

Confocal imaging was done with a Leica SP8 scanning laser microscope (Leica) using sequential, resonant scan mode. Sequential scans strategy with laser lines and detectors settings for maximal sensitivity without spectral overlap are described in Supplementary Table II. Images were acquired in tile scan mode at an optical resolution of 1024 x 1024, and tiles were stitched using the mosaic merge function in LASX software (Leica). Whole-mounted carotids and 100 µm cryosections were imaged with a 20x oil immersion objective (NA = 0.75) and 14 µm cryosections with a 40x oil immersion objective (NA = 1.3). The distance between sections in Z-stacks was 5-6 µm for whole-mounted carotid arteries, 5 µm for tissue explants, 3-4 µm for thick cryosections and 3 µm for thin cryosections. Z-compensation (laser power) was used to normalize fluorescent protein intensity throughout the tissue in thick specimens.

Image analysis was done using Imaris software (9.0.2) to adjust brightness and contrast, generate maximal projections, for virtual cross sectioning, surface rendering and measurements. Immunostained sections were imaged in the same session as control IgG samples, with identical microscope settings and image processing. The contrast and brightness were adjusted such that no signal was detected in the isotype control slide and a positive control samples was not over-saturated.

In ligated carotid arteries, regions containing ≥3 adjacent cells of the same color were scored as a medial patch if the distance between the edges of neighboring cells was less than 0.5 µm (CFP), 1.0 µm (RFP, YFP) or 15 µm (GFP) and did not cross an elastic laminal layer. Patch size was scored as small (3-10 cells), medium (11-50 cells) or large (>50 cells) manually. Bulged regions were scored as continuous stretches with outer elastic lamina diameter ≥450 µm. Intimal patches were scored as contiguous regions with ≥5 cells of the same color inside of the inner elastic lamina. The lengths of the bulged regions were measured as the length of the outer elastic lamina. Acellular (DAPI-Confetti-) medial areas were outlined in virtual cross sections using the *Measurement Points* tool in Imaris. For each artery, the percentage acellularity relative to total medial area was calculated for 5 sections in a region with normal diameter and 5 sections in a "bulged" region (if present). Arteries where image quality was affected by tissue damage or autofluorescence (8 arteries) or arteries analyzed more than 10 days after injury but did not show any sign of reaction to surgery (e.g. adventitial expansion, 3 arteries) were excluded from analysis.

VSMC plaque contribution was assessed by scoring the number of Confetti reporter colors, the total number of Confetti+ cells and the positions of Confetti+ cells in 100 µm plaque sections spaced approximately 100 µm apart along the arterial tree including the aortic root, ascending aorta, the apex of the aortic arch, carotid arteries and descending aorta. Where a plaque spanned multiple 100 µm sections, the highest scoring section with regard to number of Confetti+ cells was used. Lesion size was scored as “very small”, “small”, “medium”, “large” or “very large”, based on a qualitative assessment of the area of the lumen each occupied.

For tissue explants, surfaces were rendered for each Confetti color using default settings for the Imaris *Surfaces* function with background subtraction (a comparison of rendered and original explant images is shown in Supplementary Figure III B, C). Rendered images were

used to calculate volumes of surfaces, with patch-calling thresholds of 30,000 μm^3 for CFP/RFP/YFP or 5,000 μm^3 for GFP.

Isolation of single-cell VSMC suspensions

Aortas or carotid arteries from wild type or VSMC lineage-labeled animals (Myh11-Confetti, Myh11-EYFP or Myh11-EYFP-Ki67) were cut open longitudinally and the endothelium removed by gentle scraping with a cotton bud before removal of the adventitia as described above. The medial layer was digested to a single-cell suspension in DMEM supplemented with 2.5 mg/mL Collagenase IV (Invitrogen) and 2.5 U/mL Elastase (Worthington) at 37 ˚C.

Flow cytometry assisted cell sorting (FACS), Flow cytometry, Image stream

Antibodies used for staining are described in Supplementary Table I. Single-cell suspensions incubated with 5 µg/mL TruStain FcX anti-mouse CD16/32 antibody (Biolegend) in FACS buffer (0.5% (w/v) BSA in PBS) for 15 minutes on ice to block Fc-receptors, incubated with primary or isotype control antibody for 15 minutes at room temperature and washed twice in FACS buffer. Where needed, cells were incubated with secondary antibody in FACS buffer for 15 minutes at room temperature and washed twice in FACS buffer. Intracellular targets (ROCK1, Proteintech, 21850-1-AP) were stained using the Foxp3 staining buffer set (eBioscience) and phalloidin-iFluor™ 350 was included with secondary antibodies. Stained cells were filtered (40 µm) and either sorted (BD FACSAria™ III, BD Bioscience) or subjected to either flow cytometry (Accuri C6 or BD Fortessa, BD Bioscience) or Imagestream (Amnis® ImageStream®XMk II, Luminex) analysis. Flow cytometry compensation was done using single stained samples and gates were defined based on samples stained with control antiserum and wild type cells.

Clonal proliferation assay

Medial cells from VSMC-lineage labeled Myh11-Confetti animals were mixed with medial cells from wild type animals in a 1:3 ratio and a total of 5,000 cells seeded per well of a 96-well imaging plate (CellCarrier-96 Ultra, Perkin Elmer) in DMEM supplemented with 10% (v/v) FBS, 100 U/mL penicillin, 100 mg/mL streptomycin and 2 mM Glutamine. Medial cells from lineage-labeled Myh11-EYFP animals stained for SCA1 and 500 SCA1+EYFP+ or SCA1-EYFP+ VSMCs were isolated by FACS and mixed with 4500 wild type medium cells before plating. Medium was changed every 2-3 days and the cells were imaged 4 days after plating and after 1, 2, 3 weeks of culture using an Opera Phenix high content screening system (Perkin Elmer). Image analysis was done using Harmony software (Perkin Elmer) and quantification was performed in Fiji[8]. Patches were defined as an area with three or more contiguous lineage-labelled cells of the same colour. To calculate the area of patches images were thresholded after enhancing local contrast (CLAHE)[9]. The "analyze particles" function was then used to generate a mask of the image, and areas of pre-identified patches were extracted.

scRNA-seq data generation

Single-cell suspensions of medial cells were generated from VSMC lineage-labeled Myh11-EYFP-Ki67/RFP animals as described above. For the D5-VSMC dataset, ligated carotid arteries from six animals were isolated 5 days after surgery and cells pooled prior to FACS isolation of EYFP+ cells. Cells (10,000) were loaded onto the Chromium system (10x Genomics) and amplified cDNA libraries generated using the 3' Gene Expression v3.0 kit (10x Genomics) were sequenced using a 150 cycle protocol (NovaSeq, Illumina). For the

D5-all arterial cell dataset, EYFP+ VSMCs were mixed with EYFP- cells and cDNA libraries prepared in parallel to the D5-VSMC sample. To generate the 10x D7 dataset, cells from the ligated left carotid arteries of five animals dissected 7 days after surgery were pooled and enriched for proliferating RFP+EYFP+ cells during FACS. A total of 100 EYFP+RFP+ cells were supplemented with 20,000 unselected EYFP+ cells before loading onto a 10x Chromium system. Amplified cDNA was generated using the 3' Gene Expression v2 kit and sequenced using a paired end protocol (HiSeq 4000, Illumina). The Smart-seq2 protocol[10] was used to generate amplified cDNA libraries from EYFP+RFP+ and EYFP+RFP- cells isolated from the ligated arteries of animals 7 days after surgery and EYFP+ cells from litter mate control animals in 2 separate experiments (4 or 5 ligated arteries and 1 or 2 control arteries were pooled respectively in the two experiments). Cells were processed as described[11] with addition of ERCC spike controls (diluted 1:80,000,000, Invitrogen). Pooled cDNA libraries were sequenced (HiSeq 2500, Illumina).

scRNA-seq data analysis

For the D7 Smart-seq2 data set, reads were aligned to the GRCm38 mouse genome using *TopHat* v2.1[12] and *Htseq-count* v0.8[13] was used to count the number of read alignments per gene in the resulting bam files (idattr = "gene_id" and stranded = "no"). Cells with <200,000 total reads, <1,000 genes detected and >30% of ERCC controls were excluded from analysis and genes with mean expression <1 were removed. The function computeSumFactors from the Bioconductor R package *scran*[14] was used to compute the normalization factors for individual cells.

The 10x Genomics Cell Ranger pipeline v.2.1.1 was used to align raw sequencing reads to GRCm38 mouse genome and count the number of reads aligning to each gene for the D7 dataset. Cell Ranger pipeline v.3.1.0 was used to align raw sequencing reads to a custom genome (based on the GRCm38 mouse genome), including the open reading frame encoding EYFP[4] for the D5-EYFP and D5-all arterial cells datasets. A previously published 10x scRNA-seq dataset of VSMC-lineage labeled plaque cells from Myh11-Confetti-Apoe animals fed a high fat diet for 14 or 18 weeks and a Smart-seq2 dataset from Myh11-Confetti animals including SCA1 positive and SCA1 negative cells expressing the VSMC lineage label and SCA1 positive cells not expressing Confetti label were obtained from the Gene Expression Omnibus (GEO) repository (accession number: GSE117963)[11]. Quality control (QC), normalization and gene expression analysis was performed using the CRAN R package *Seurat* v.3.1.2[15,16] in R v.3.6.2. Cells with <5,000 UMI counts, <2,000 genes detected and >5% mitochondrial reads were excluded for the D5-EYFP dataset, cells with <1,900 genes detected and >5% mitochondrial reads were excluded for the D5-all arterial cells, and for the D7 dataset cells with <5,000 UMI counts, <2,000 genes detected and >6% mitochondrial reads were excluded. For the plaque dataset, cells with <5,000 or >20,000 UMI counts, <1,000 or >5,000 genes detected and >9% mitochondrial reads were excluded from the analysis. Normalization was performed using *SCTransform*[17] v.0.2.1 implemented in *Seurat*. Principal component analysis (PCA) was performed with 3,000 highly variable genes (HVG). The first 27 (D5-EYFP), 30 (D5-all arterial cells), 25 (D7), or 30 (plaque) principal components (PCs) were used, and clustering was done at resolution 0.8 (D5-EYFP and D5-all arterial cells), 1.6 (D7), or 1.1 (plaque).

Cell cluster marker genes in the D7 dataset were identified through differential expression testing via Wilcoxon rank sum test (FindAllMarkers, log fold-change > 0.5, adjusted p-value < 0.05) and visualized using the DoHeatmap function in *Seurat*. Data integration (D7 Smart-

seq2 and D7 10x datasets, or D5-all arterial cells and SCA1 Smart-seq2 datasets) was done using *Seurat* v.3.2.1[15]. Prior to integration, genes expressed in less than 3 cells and cells with high mitochondrial reads were removed from the Smart-seq2 datasets (>15% for D7 post injury and >25 for cells from healthy arteries). Each dataset was log normalized, and 2,000 HVGs were identified in each prior to integration. The first 30 PCs were used for UMAP visualization of the integrated datasets.

Trajectory inference was performed using the Bioconductor R package *slingshot* v.1.4.0[18] after the analysis described above or with partition-based graph abstraction (PAGA) in *ScanPy*[19] using all genes as input (connectivity threshold 0.5). For the D5 slingshot analysis, cluster 1 was used as the starting cluster. Significance of differential expression along pseudotime was tested through CRAN R package *gam* v.1.16.1 with an FDR-adjusted p-value threshold of 0.05, and a magnitude threshold of 0.5 (log scale) was applied. Genes showing differential expression along pseudotime were hierarchically clustered using CRAN R package *pheatmap* v.1.0.12 with the Pearson correlation distance and complete linkage method. The optimal number of gene clades was determined through the elbow method which was performed via CRAN R package *factoextra* v.1.0.7.

Gene ontology (GO) term analysis of Path1 gene clusters was performed with the enrichGO and compareCluster functions implemented in Bioconductor R package *clusterProfiler* v.3.14.3[20] using genome-wide annotation for mouse with Bioconductor R package *org.Mm.eg.db* v.3.10.0. with multiple testing correction (Benjamini-Hochberg) and p-value and q-value thresholds of 0.05.

The summarized expression level of gene subsets was calculated through their first principal components across the cells in the respective dataset and the Module Score calculated using the AddModuleScore function in *Seurat*. The gene list of the VSMC response signature was obtained from Dobnikar et al.[11]. Path1-induced genes included gene clades 1, 2, 3, 4, 8.

Supplementary References

1. Chakraborty R, Saddouk FZ, Carrao AC, Krause DS, Greif DM, Martin KA. Promoters to Study Vascular Smooth Muscle. *Arteriosclerosis Thrombosis Vasc Biology* 2019;39:603–612.

2. Wirth A, Benyó Z, Lukasova M, Leutgeb B, Wettschureck N, Gorbey S, Örsy P, Horváth B, Maser-Gluth C, Greiner E, Lemmer B, Schütz G, Gutkind JS, Offermanns S. G12-G13–LARG–mediated signaling in vascular smooth muscle is required for salt-induced hypertension. *Nat Med* 2008;14:64–68.

3. Snippert HJ, Flier LG van der, Sato T, Es JH van, Born M van den, Kroon-Veenboer C, Barker N, Klein AM, Rheenen J van, Simons BD, Clevers H. Intestinal Crypt Homeostasis Results from Neutral Competition between Symmetrically Dividing Lgr5 Stem Cells. *Cell* 2010;143:134–144.

4. Srinivas S, Watanabe T, Lin C-S, William CM, Tanabe Y, Jessell TM, Costantini F. Cre reporter strains produced by targeted insertion of EYFP and ECFP into the ROSA26 locus. *Bmc Dev Biol* 2001;1:4.

5. Basak O, Born M, Korving J, Beumer J, Elst S, Es JH, Clevers H. Mapping early fate determination in Lgr5+ crypt stem cells using a novel Ki67-RFP allele. *Embo J* 2014;33:2057–2068.

6. Piedrahita JA, Zhang SH, Hagaman JR, Oliver PM, Maeda N. Generation of mice carrying a mutant apolipoprotein E gene inactivated by gene targeting in embryonic stem cells. *Proc National Acad Sci* 1992;89:4471–4475.

7. Chappell J, Harman JL, Narasimhan VM, Yu H, Foote K, Simons BD, Bennett MR, Jørgensen HF. Extensive Proliferation of a Subset of Differentiated, yet Plastic, Medial Vascular Smooth Muscle Cells Contributes to Neointimal Formation in Mouse Injury and Atherosclerosis Models. *Circ Res* 2016;119:1313–1323.

8. Schindelin J, Arganda-Carreras I, Frise E, Kaynig V, Longair M, Pietzsch T, Preibisch S, Rueden C, Saalfeld S, Schmid B, Tinevez J-Y, White DJ, Hartenstein V, Eliceiri K, Tomancak P, Cardona A. Fiji: an open-source platform for biological-image analysis. *Nat Methods* 2012;9:676–682.

9. Zuiderveld K. Contrast Limited Adaptive Histogram Equalization. *Graphics Gems* 1994:474--485.

10. Picelli S, Faridani OR, Björklund ÅK, Winberg G, Sagasser S, Sandberg R. Full-length RNA-seq from single cells using Smart-seq2. *Nat Protoc* 2014;9:171–181.

11. Dobnikar L, Taylor AL, Chappell J, Oldach P, Harman JL, Oerton E, Dzierzak E, Bennett MR, Spivakov M, Jørgensen HF. Disease-relevant transcriptional signatures identified in individual smooth muscle cells from healthy mouse vessels. *Nat Commun* 2018;9:4567.

12. Trapnell C, Pachter L, Salzberg SL. TopHat: discovering splice junctions with RNA-Seq. *Bioinformatics* 2009;25:1105–1111.

13. Anders S, Pyl PT, Huber W. HTSeq—a Python framework to work with high-throughput sequencing data. *Bioinformatics* 2015;31:166–169.

14. Lun ATL, McCarthy DJ, Marioni JC. A step-by-step workflow for low-level analysis of single-cell RNA-seq data with Bioconductor. *F1000research* 2016;5:2122.

15. Stuart T, Butler A, Hoffman P, Hafemeister C, Papalexi E, Mauck WM, Hao Y, Stoeckius M, Smibert P, Satija R. Comprehensive Integration of Single-Cell Data. *Cell* 2019;177:1888-1902.e21.

16. Butler A, Hoffman P, Smibert P, Papalexi E, Satija R. Integrating single-cell transcriptomic data across different conditions, technologies, and species. *Nat Biotechnol* 2018;36:411–420.

17. Hafemeister C, Satija R. Normalization and variance stabilization of single-cell RNA-seq data using regularized negative binomial regression. *Genome Biol* 2019;20:296.

18. Street K, Risso D, Fletcher RB, Das D, Ngai J, Yosef N, Purdom E, Dudoit S. Slingshot: cell lineage and pseudotime inference for single-cell transcriptomics. *Bmc Genomics* 2018;19:477.

19. Wolf FA, Hamey FK, Plass M, Solana J, Dahlin JS, Göttgens B, Rajewsky N, Simon L, Theis FJ. PAGA: graph abstraction reconciles clustering with trajectory inference through a topology preserving map of single cells. *Genome Biol* 2019;20:59.

20. Yu G, Wang L-G, Han Y, He Q-Y. clusterProfiler: an R Package for Comparing Biological Themes Among Gene Clusters. *Omics J Integr Biology* 2012;16:284–287.

21. Pan H, Xue C, Auerbach BJ, Fan J, Bashore AC, Cui J, Yang DY, Trignano SB, Liu W, Shi J, Ihuegbu CO, Bush EC, Worley J, Vlahos L, Laise P, Solomon RA, Connolly ES, Califano A, Sims PA, Zhang H, Li M, Reilly MP. Single-Cell Genomics Reveals a Novel Cell State During Smooth Muscle Cell Phenotypic Switching and Potential Therapeutic Targets for Atherosclerosis in Mouse and Human. *Circulation* 2020;142:2060–2075.

22. Gao X-F, Chen A-Q, Wang Z-M, Wang F, Luo S, Chen S-Y, Gu Y, Kong X-Q, Zuo G-F, Chen Y, Ge Z, Zhang J-J, Chen S-L. Single-Cell RNA Sequencing of the Rat Carotid Arteries Uncovers Potential Cellular Targets of Neointimal Hyperplasia. *Frontiers Cardiovasc Medicine* 2021;8:751525.

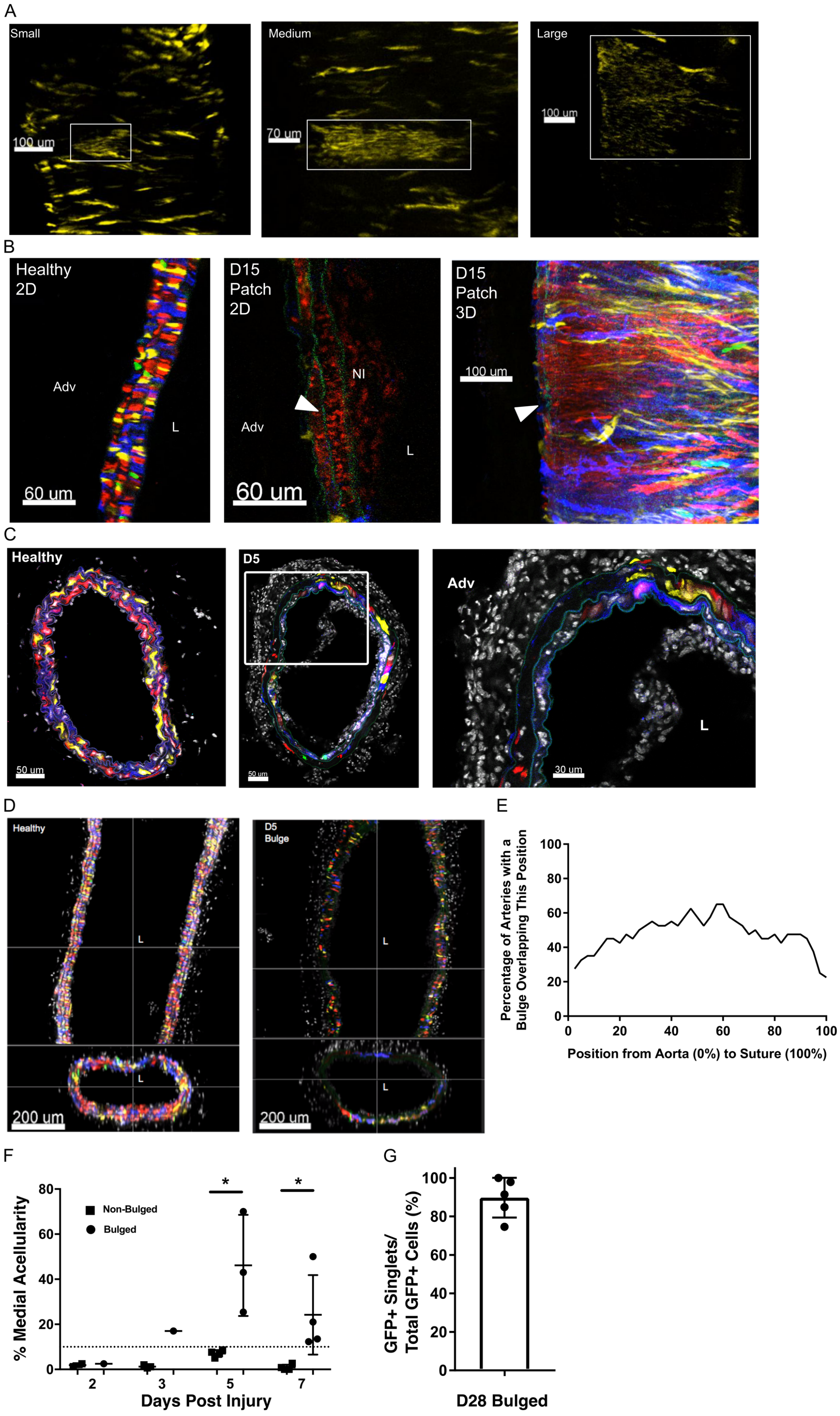

Supplementary Figure I: Examples and quantification of whole-mounted left carotid arteries of VSMC-lineage labeled Myh11-Confetti animals. A, Confocal images (max.

projections) of ligated carotid arteries. Boxes show examples of small (left), medium (middle) and large VSMC patches (right). Only signals from the yellow fluorescent protein (EYFP) of the Confetti reporter are shown. Scale bars = 100 μm (left and right panels), 70 μm (middle). **B,** Single Z-scans of longitudinal carotid artery Z-stack from unligated control animal (left) and ligated artery 15 days (D15) after surgery (middle) are shown to illustrate cellular disarray in regions with medial VSMC patches (RFP+, arrowhead). A neointimal (NI) RFP+ patch is also present. The adventitial (Adv) and luminal (L) sides are indicated. Scale bars = 60 μm. Right panel shows max. projection of the D15 artery for comparison, with arrowhead pointing to the RFP+ medial VSMC patch shown in the middle panel. Scale bar = 100 μm. **C,** Cryosections of carotid arteries analyzed 5 days after ligation (left) or from unligated animal (middle; right panel shows magnified view of the boxed region). Scale bar = 50 μm. Signals for Confetti and DAPI (white) are shown. **D,** Virtual horizontal (top) and transverse (lower) cross-sections generated in Imaris from whole mount images of carotid arteries from an unligated control animal (left) and a bulged region from a ligated artery 5 days (D5) after surgery (right). Signals for DAPI (white) and the Confetti reporter are shown. Scale bars = 200 μm. **E,** Graph shows the percentage of analysed ligated carotid arteries containing a bulged region overlapping each position along the artery from the aorta (0%) to the ligation suture (100%). **F**, Bar graph showing the percentage of medial area without Confetti and DAPI signal in virtual cross-sections from whole mounted arteries 2-7 days after ligation. Dots show mean of 3-5 cross-sections for each animal in bulged (circles) or non-bulged regions (squares). For each group, the mean and standard deviation are indicated. Asterisks indicate $P<0.05$, Mann-Whitney U test. **G,** The fraction of GFP+ cells which are singlets (rather than in patches) was quantified in bulged regions of carotid arteries analysed at Day 28 (D28) post-ligation. Values for individual arteries (n=5), mean and standard deviation are shown.

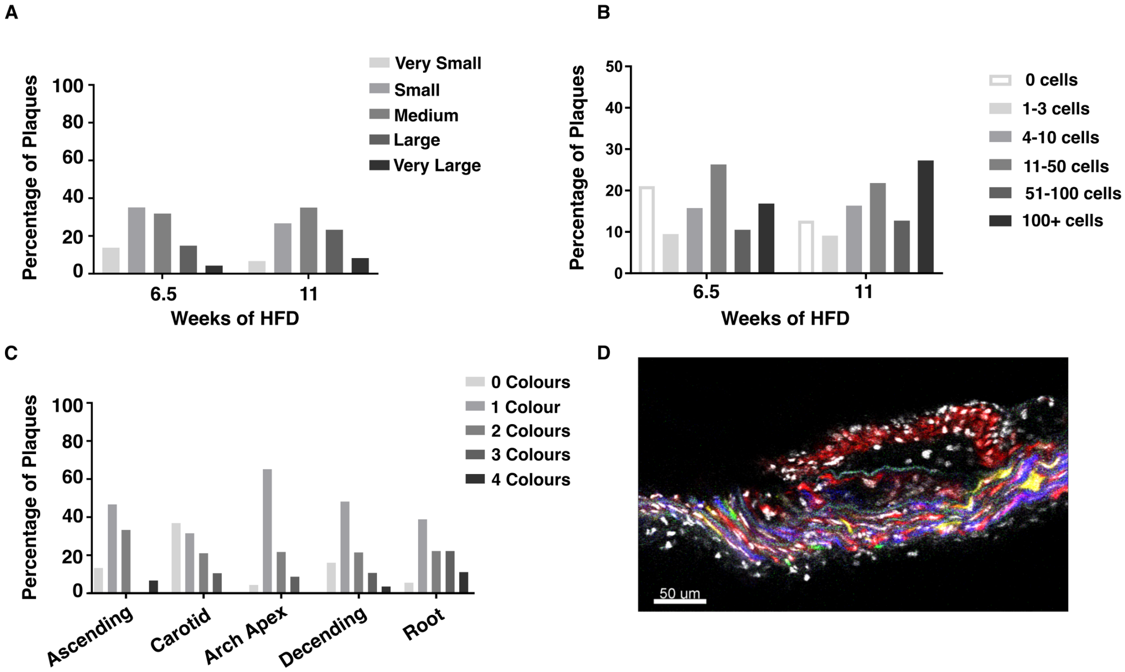

Supplementary Figure II: Quantification of plaques in VSMC-lineage labeled Myh11-Confetti-Apoe animals. A, Plaque size distribution in animals analyzed after 6.5 weeks (95 lesions in 4 animals) or 11 weeks (55 lesions in 3 animals) high fat diet, HFD. **B,** Distribution of number of Confetti+ cells within plaques at 6.5 and 11 weeks of HFD**. C,** Percentage of plaques where 0, 1, 2, 3, 4 Confetti colors were detected, stratified by vascular region (ascending = 15, carotid = 40, arch apex = 23, descending = 54, root = 18). **D,** Representative confocal image (single Z-plane) of plaque from high fat diet (HFD)-fed, VSMC-lineage traced Myh11-Confetti-Apoe animal, illustrating a medial patch, with associated medial disarray, underlying a plaque invested by VSMCs of the same color as the plaque.

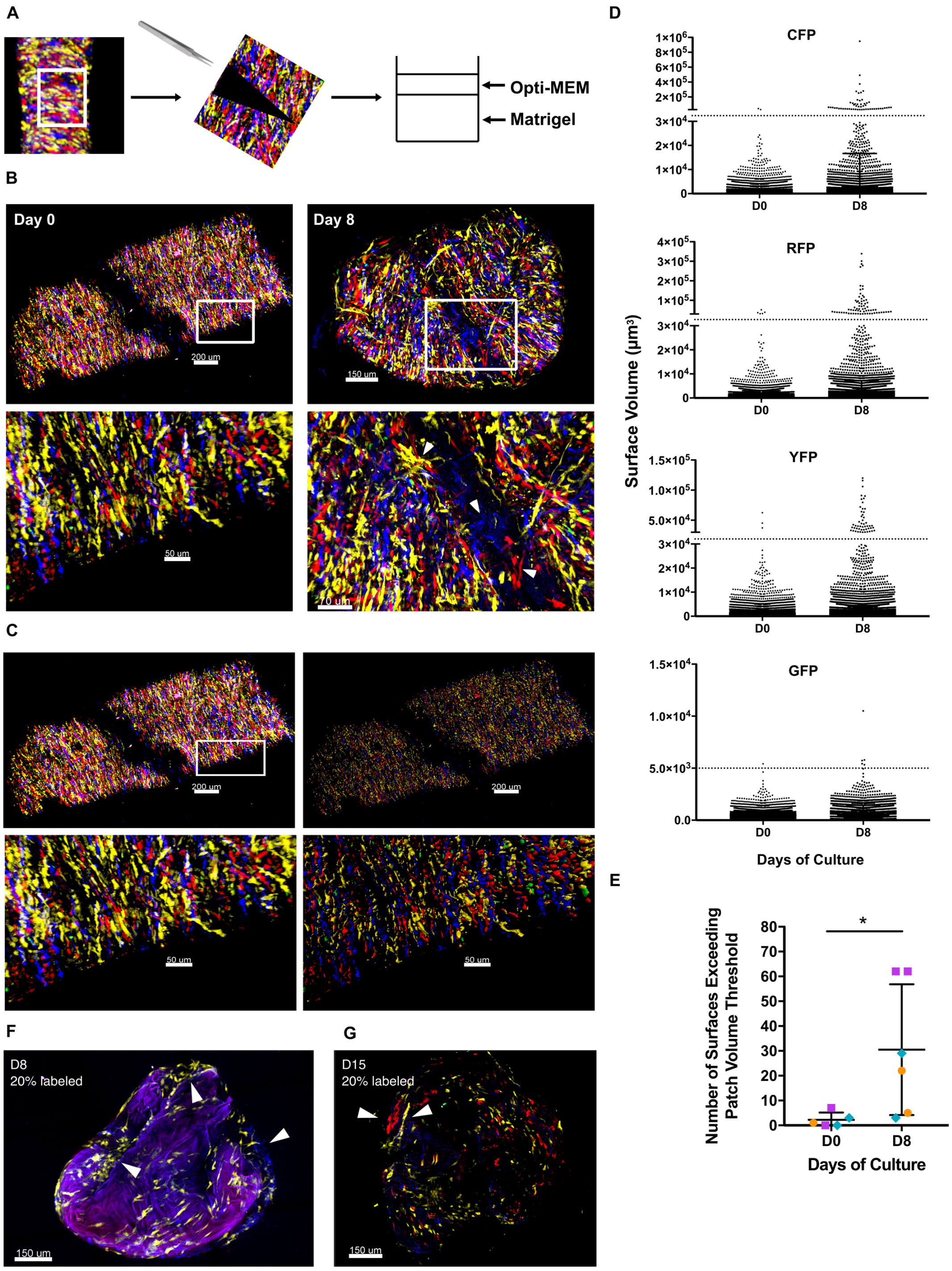

Supplementary Figure III: Rare activation of VSMC proliferation in tissue explant cultures. A, Schematic of tissue explant forceps-pinch injury-assay. **B,** Confocal images (max. projection) of tissue explants before (Day 0, left) or after (Day 8, right) culture showing Confetti signals (CFP:blue, RFP:red, YFP:yellow, GFP:green). Magnified views of boxed regions are shown in lower panels, where arrowheads point to monochromatic VSMC patches within or adjacent to forceps pinch injury. Scale bar = 200 µm (top left), 150 µm (top right), 50 µm (lower left), 70 µm (lower right). **C,** Surface rendering of confocal images of an aortic tissue explant analyzed at day 0. Signals for Confetti colors are shown and lower panels show magnified view of boxed region. Original images (left) and reconstructions after surface-rendering (right) are shown. Scale bar = 200 µm (top) or 50 µm (lower panels). **D-E,**

Quantification of surface-rendered volume of Confetti+ regions in tissue explants before (D0) or after (D8) culture. **D**, Volumes of individual surfaces are shown separately for each Confetti protein. Threshold volumes for patch calling are indicated by dotted lines. **E,** The number of surfaces exceeding patch-volume thresholds is shown for each explant. Symbols and colors show explants from different animals. Mean and standard deviation are indicated. Three animals (1-2 explants per time point from each animal) were analyzed. Asterisks indicate $P<0.05$, Welch's t-test. **F, G**, Confocal images of tissue explants from Myh11-Confetti animals with lower frequency of VSMC-lineage labeling (20%, 2 injections of 0.1 mg tamoxifen) after culture for 8 (**F**, max. projection) or 15 days (**G**, single Z-scan). Arrows point to mono-chromatic VSMC patches. Scale bar = 150 μm.

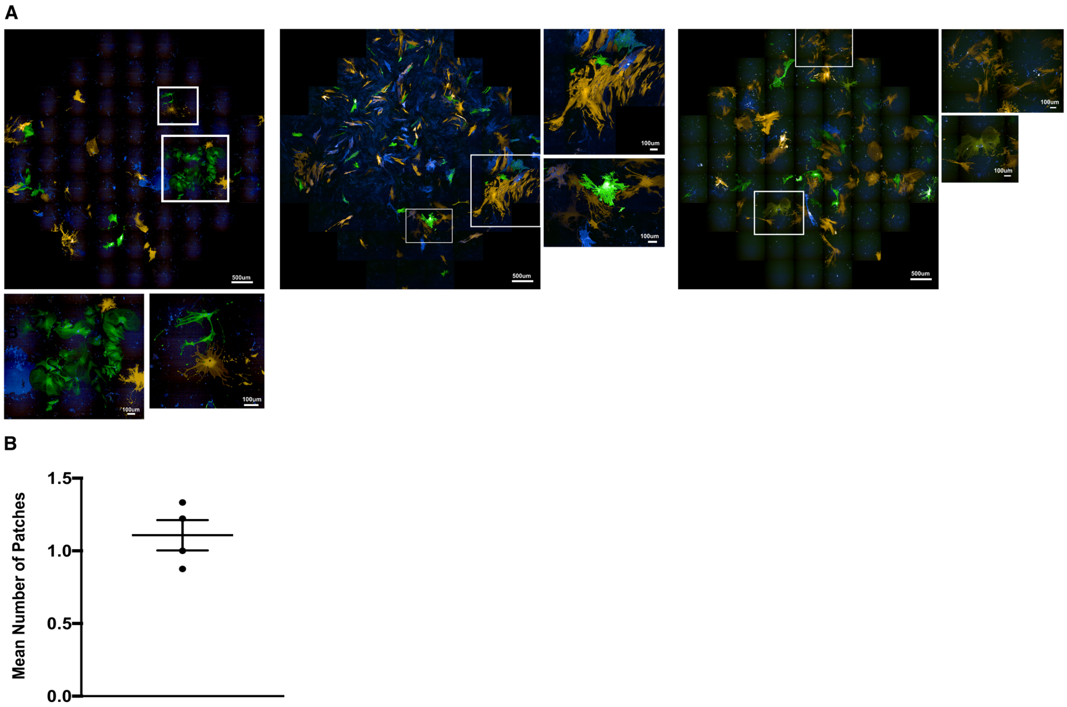

Supplementary Figure IV: Clonal proliferation of dissociated VSMCs in culture. A, Examples of live cell images (Opera Phenix) of VSMCs from Myh11-Confetti and wild type animals (mixed 1:3) after 3 weeks of culture. Magnified views of boxed regions with representative VSMC patch or non-expanded singlet Confetti+ cells is shown for each example. Signals for Confetti proteins are shown (GFP or YFP: green, RFP: orange, CFP: blue). Scale bar = 500 μm, 100 μm (magnified views). **B,** Quantification of number of Confetti+ patches per well. Dots show mean of 3 wells per animal. Mean and S.E.M. from 4 animals, analyzed separately, are shown.

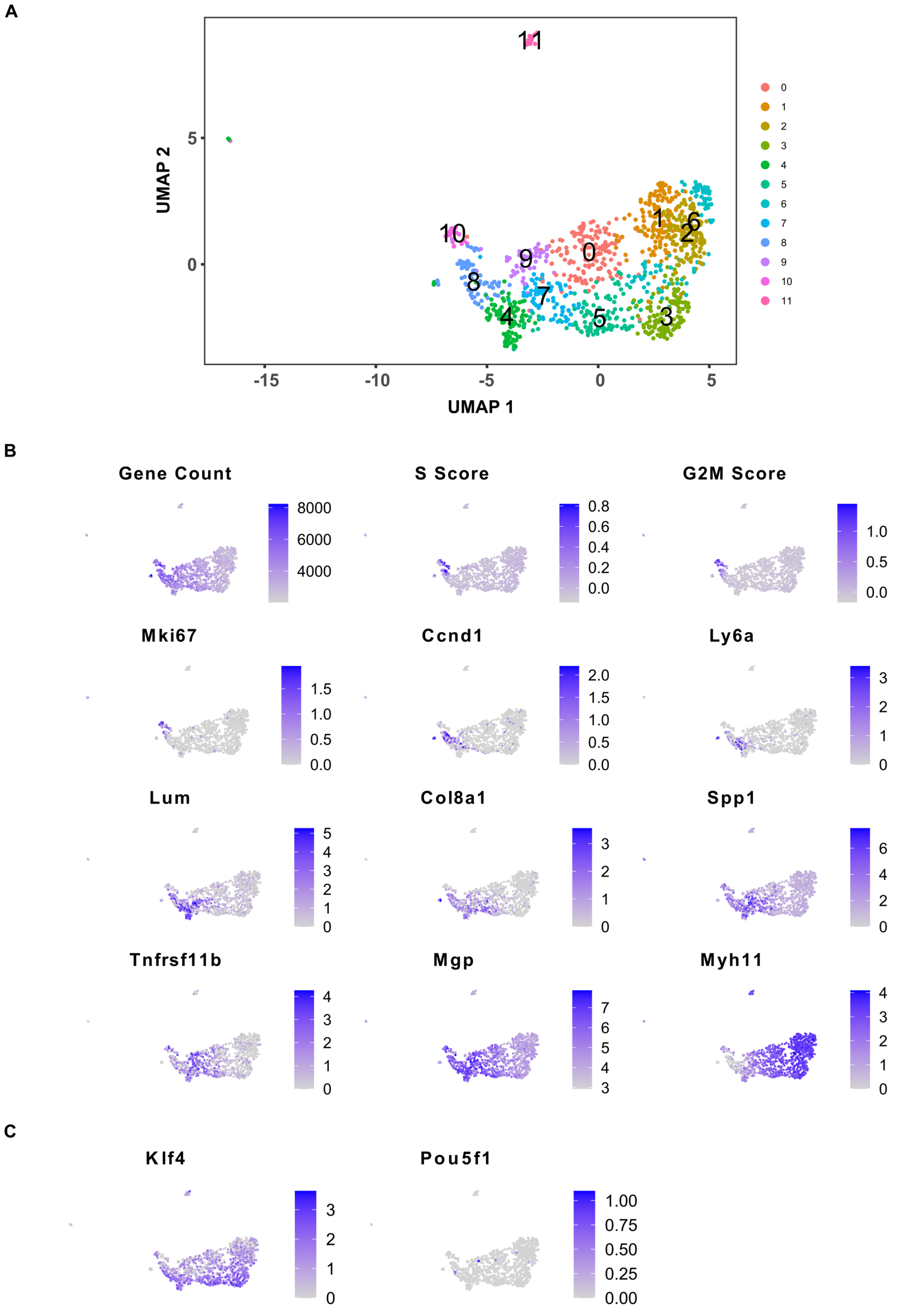

Supplementary Figure V: Feature plots of mouse D7 scRNA-seq dataset including cell cluster 11. UMAP showing cell clusters **(A)** and feature plots **(B)** corresponding to Figure 5A, B including cluster 11 cells. Cluster 11 markers include *Prss12*, *Prrx2*, *Rgs5* similar to the "minor SMC" population described by Pan *et al.* (Pan et al., 2020)[21]. **C,** Feature plots showing expression of VSMC regulators *Klf4* and *Oct4* (*Pou5f1*) in a grey-blue scale.

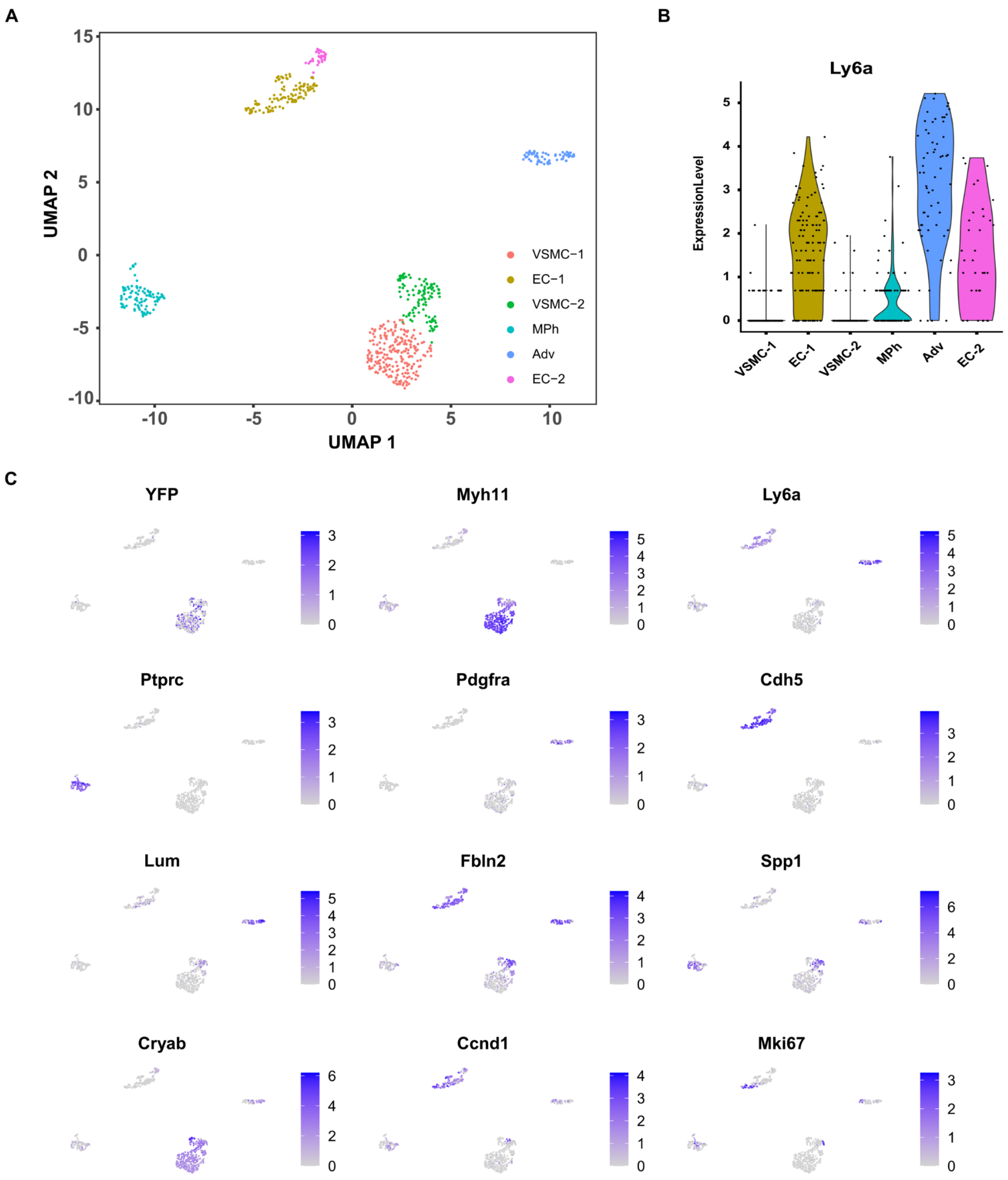

Supplementary Figure VI: VSMCs form a single cell population distinct from macrophages, endothelial and adventitial cells at early timepoints (5 days) after injury. scRNA-seq analysis of all arterial cells (655 cells total) 5 days post carotid ligation. **A,** UMAP showing annotated cell populations. **B,** Violin plot showing expression of Ly6a across cell clusters. **C,** Feature plots with grey-to-blue scale showing expression of VSMC-lineage label (YFP), cell type markers (Myh11: VSMC, Ptprc: immune cells, Pdgfra: adventitial cells, Cdh5: endothelial cells) and genes associated with injury-induced VSMC modulation (Lum, Fbln2, Spp1, Cryab) and proliferation (Ccnd1, Mki67). Whereas VSMCs have been reported to adopt alternative cell states at later time points in a rat injury model[22], this was not evident here.

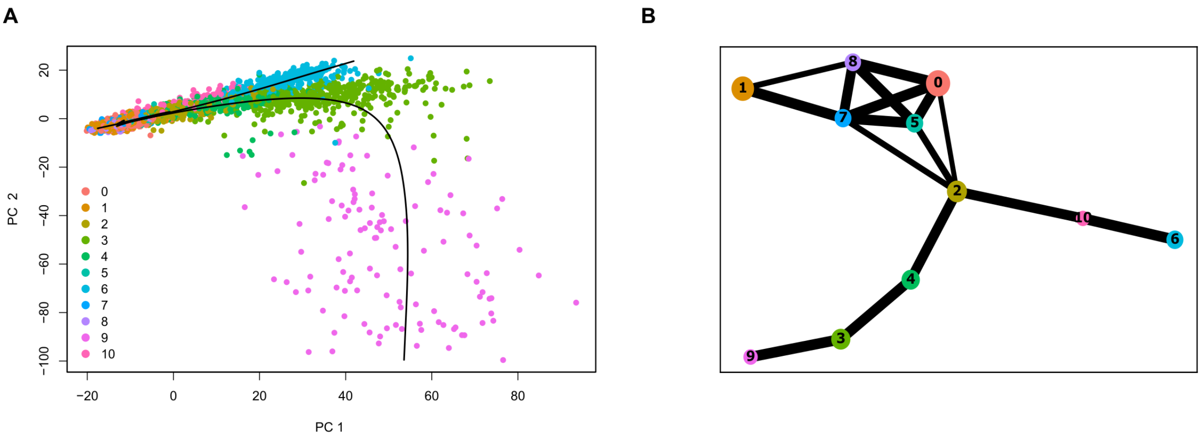

Supplementary Figure VII: Trajectory inference for mouse D5 scRNA-seq data. Trajectory inference analyses of mouse D5 scRNA-seq dataset from VSMC-lineage label+ cells from Myh11-Confetti animals 5 days after carotid ligation (mouse D5 scRNA-seq dataset) using the slingshot package (A) or partition-based graph abstraction (PAGA, B). Cluster numbers and color-coding refer to clustering shown in Figure 5A of the main manuscript and are also indicated in panel A. **A,** Plot of Principal components 1 and 2 (PC1, PC2) where each dot represents a single cell. Black lines indicate slingshot-identified paths, Path1: cluster 1 to cluster 9, and Path2: cluster 1 to cluster 6. **B,** PAGA connectivity map where black lines show connections between individual clusters and line thickness indicates the strength of the connection (threshold=0.5).

A

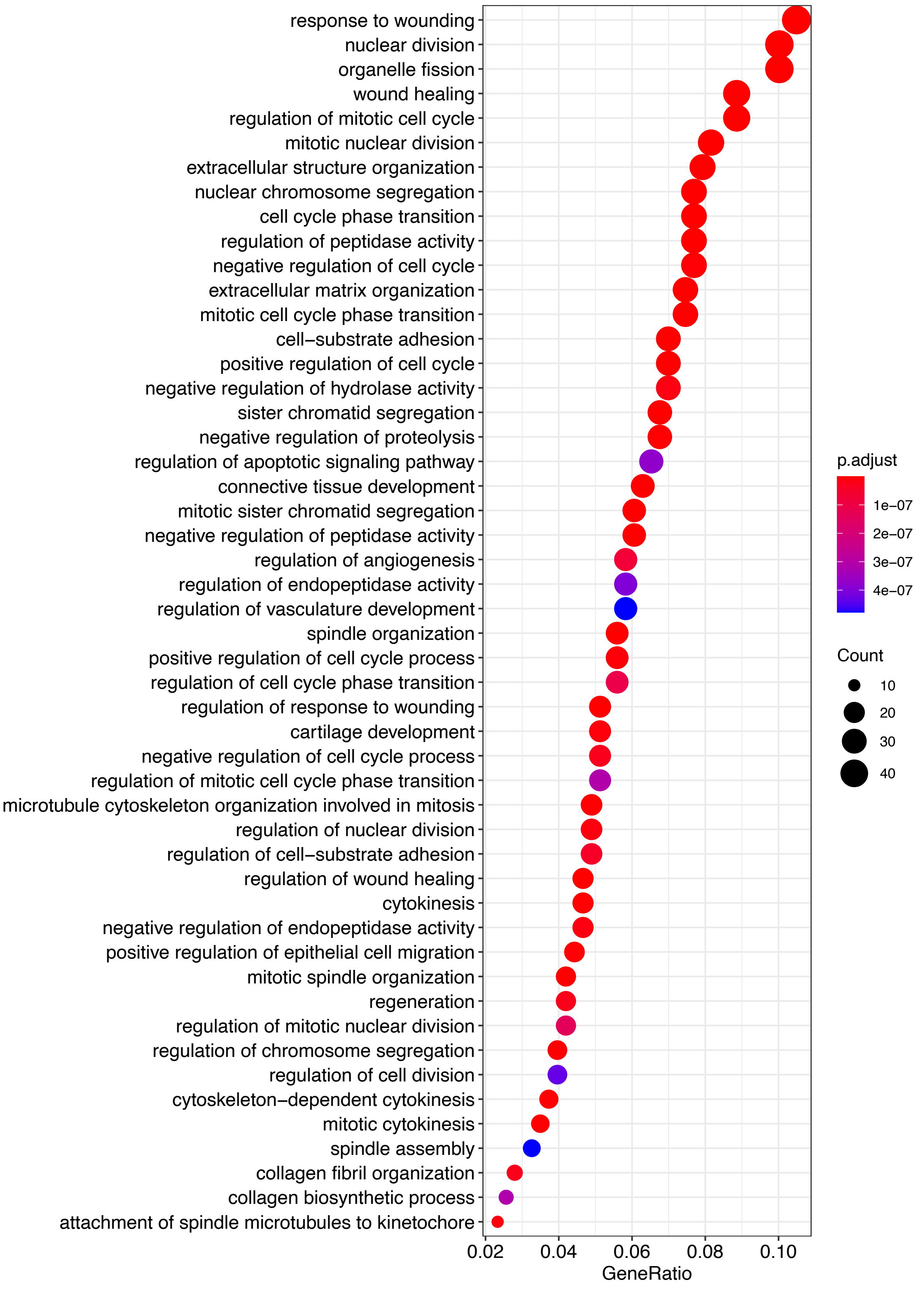
response to wounding
nuclear division
organelle fission
wound healing
regulation of mitotic cell cycle
mitotic nuclear division
extracellular structure organization
nuclear chromosome segregation
cell cycle phase transition
regulation of peptidase activity
negative regulation of cell cycle
extracellular matrix organization
mitotic cell cycle phase transition
cell−substrate adhesion
positive regulation of cell cycle
negative regulation of hydrolase activity
sister chromatid segregation
negative regulation of proteolysis
regulation of apoptotic signaling pathway
connective tissue development
mitotic sister chromatid segregation
negative regulation of peptidase activity
regulation of angiogenesis
regulation of endopeptidase activity
regulation of vasculature development
spindle organization
positive regulation of cell cycle process
regulation of cell cycle phase transition
regulation of response to wounding
cartilage development
negative regulation of cell cycle process
regulation of mitotic cell cycle phase transition
microtubule cytoskeleton organization involved in mitosis
regulation of nuclear division
regulation of cell−substrate adhesion
regulation of wound healing
cytokinesis
negative regulation of endopeptidase activity
positive regulation of epithelial cell migration
mitotic spindle organization
regeneration
regulation of mitotic nuclear division
regulation of chromosome segregation
regulation of cell division
cytoskeleton−dependent cytokinesis
mitotic cytokinesis
spindle assembly
collagen fibril organization
collagen biosynthetic process
attachment of spindle microtubules to kinetochore
0.02
0.04
0.06
0.08
0.10
GeneRatio
p.adjust
1e−07
2e−07
3e−07
4e−07
Count
10
20
30
40

B

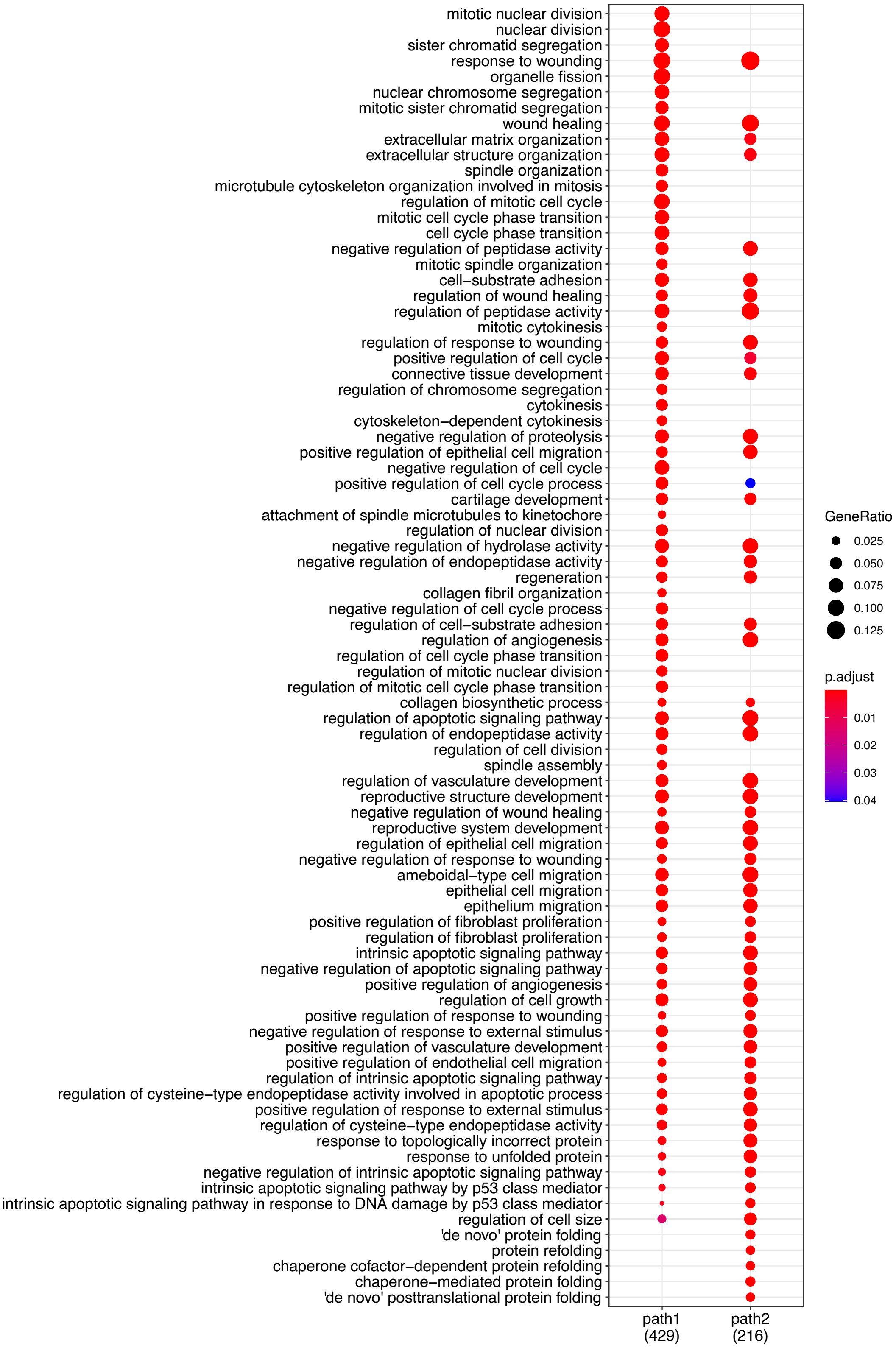

Supplementary Figure VIII: Gene ontology (GO) analysis of genes showing trajectory-induced expression in mouse D5 scRNA-seq dataset. Selected enriched GO-terms for genes showing induced expression along path1 (gene clades 1, 2, 3, 4, 8) **(A)** and induced expression along path1 (gene clades 1, 2, 3, 4, 8) versus path 2 (clade 1) **(B).**

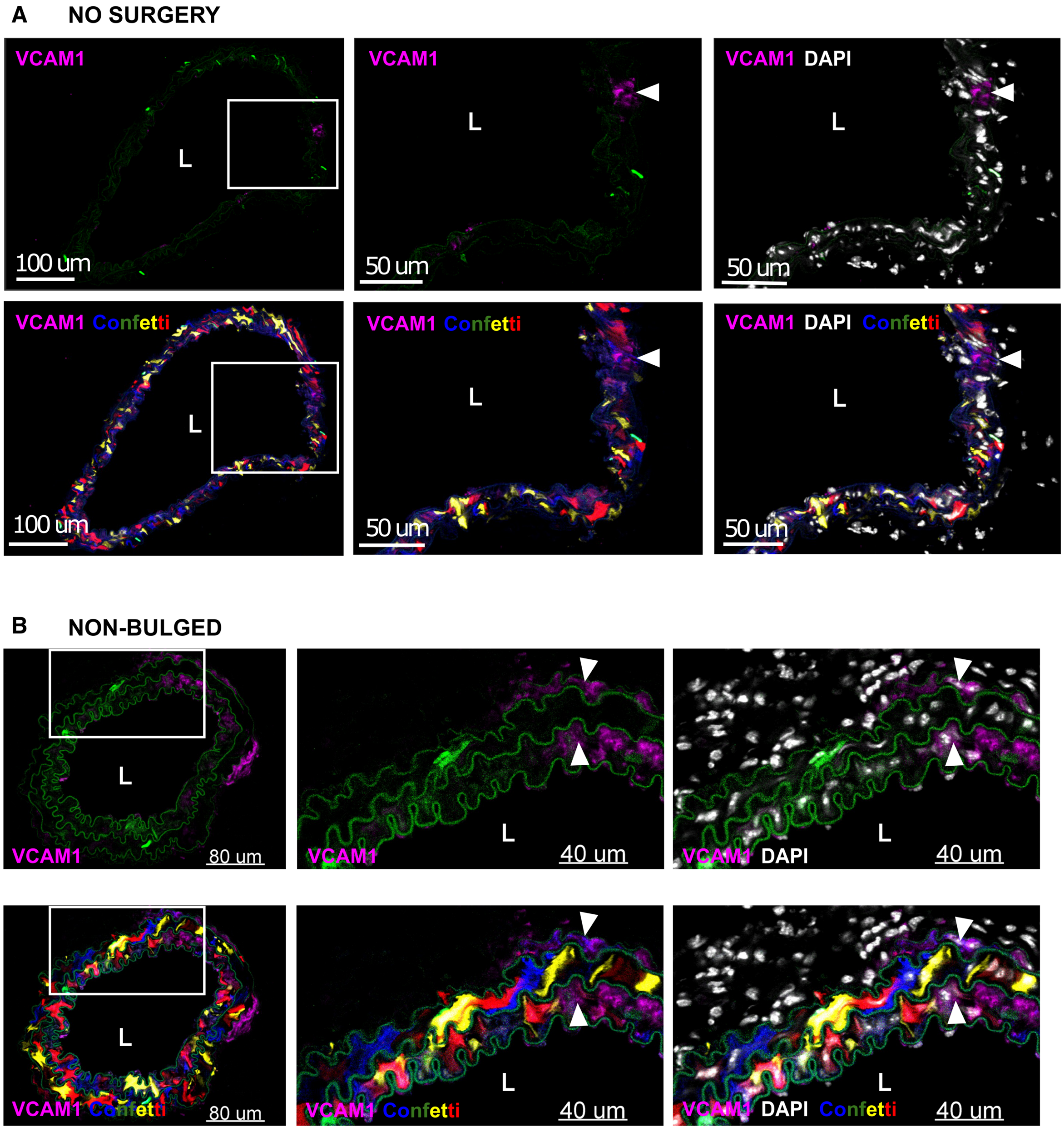

Supplementary Figure IX: Immunostaining for VCAM1. Representative immunofluorescence staining for VCAM1 in cryosections from healthy arteries (n=5 animals, average of 4 sections per animal) (**A**) and from non-bulged regions (n=5 animals, average of 4 sections per animal) of ligated carotid arteries (day 5-7 post-carotid ligation) (**B**) from VSMC lineage-labeled Myh11-Confetti animals. The panels at the left show entire arteries while middle and right panels show magnified views of the boxed regions. Signals for VCAM1 (magenta), Confetti proteins and DAPI (white) are shown as indicated. The lumen (L) is indicated. Scale bars are shown on each panel.

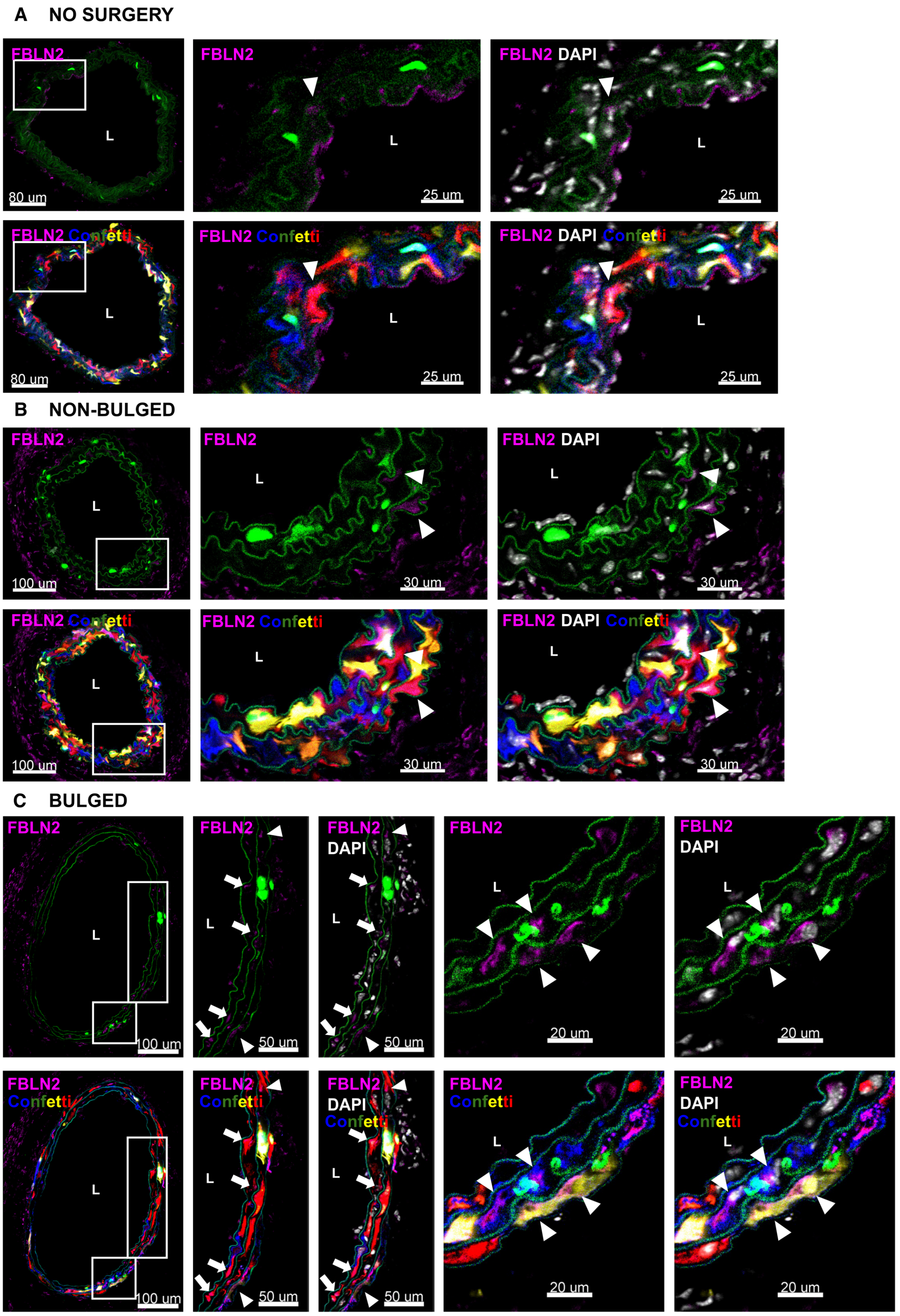

Supplementary Figure X: Immunostaining for FBLN2. Representative immunofluorescence staining for FBLN2 in cryosections from healthy arteries (n=5 animals, average of 4 sections per animal) (**A**) and from either non-bulged (**B**) or bulged (**C**) regions of ligated carotid arteries (day 5-7 post-carotid ligation, n=4 animals, average of 4 non-bulged and 4 bulged sections per animal) from VSMC lineage-labeled Myh11-Confetti animals. The panels at the left show the entire arteries, while panels to the right show magnified views of the boxed regions. Signals for FBLN2 (magenta), Confetti proteins and DAPI (white) are shown as indicated. Arrows point to FBLN2+ cells in the patches and arrowheads point to FBLN2+ cells in VSMC singlets. The lumen (L) is indicated. Scale bars are shown on each panel.

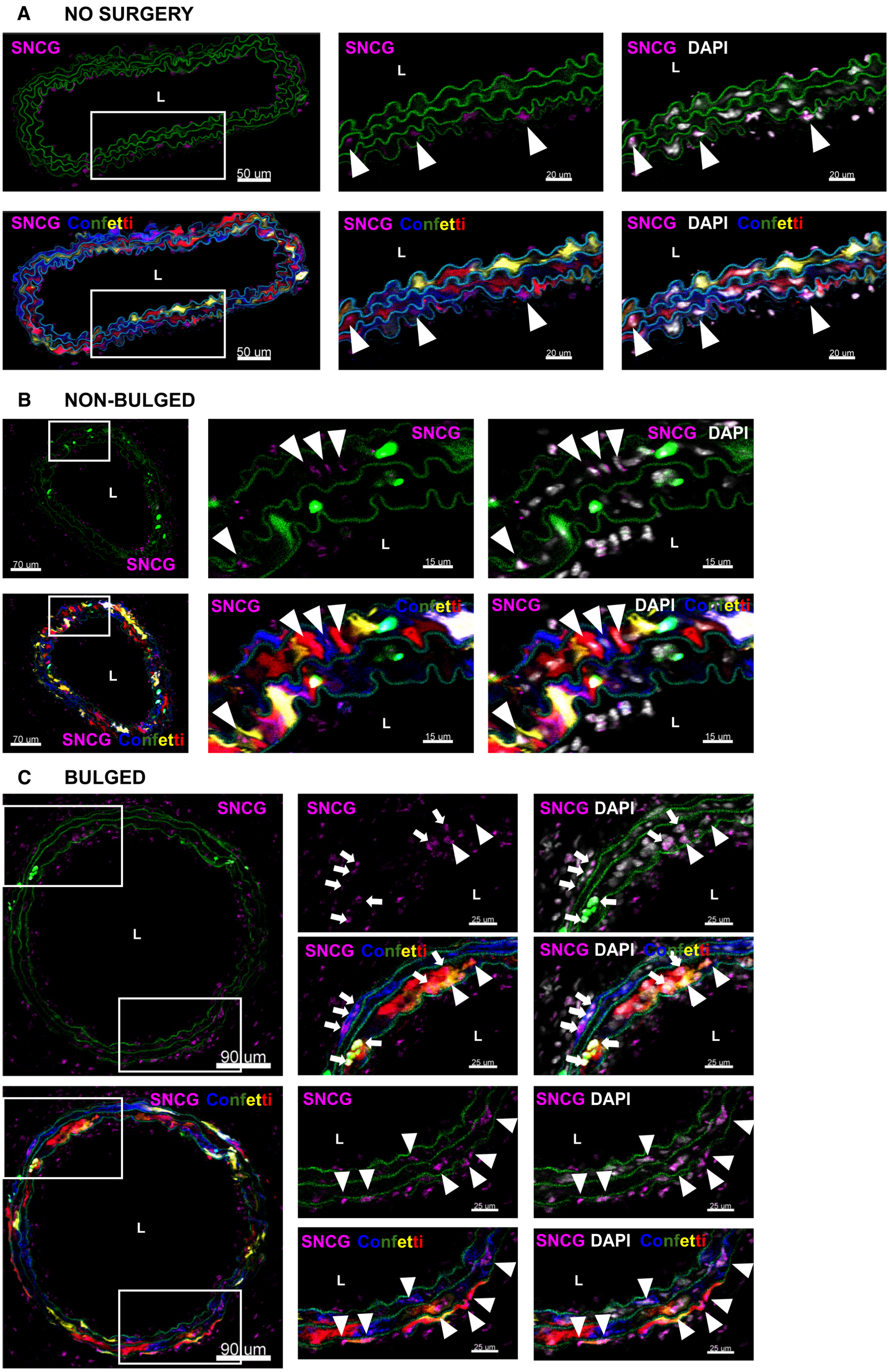

Supplementary Figure XI: Immunostaining for SNCG. Representative immunofluorescence staining for SNCG in cryosections from healthy arteries (n=5 animals, average of 4 sections per animal) (**A**) and from either non-bulged (**B**) or bulged (**C**) regions

of ligated carotid arteries (day 5-7 post-carotid ligation, n=4 animals, average of 4 non-bulged and 4 bulged sections per animal) from VSMC lineage-labeled Myh11-Confetti animals. The panels at the left show the entire arteries, while panels to the right show magnified views of the boxed regions. Signals for SNCG (magenta), Confetti proteins and DAPI (white) are shown as indicated. Arrows point to SNCG+ cells in patches and arrowheads point to SNCG+ cells in VSMC singlets. The lumen (L) is indicated. Scale bars are shown on each panel.

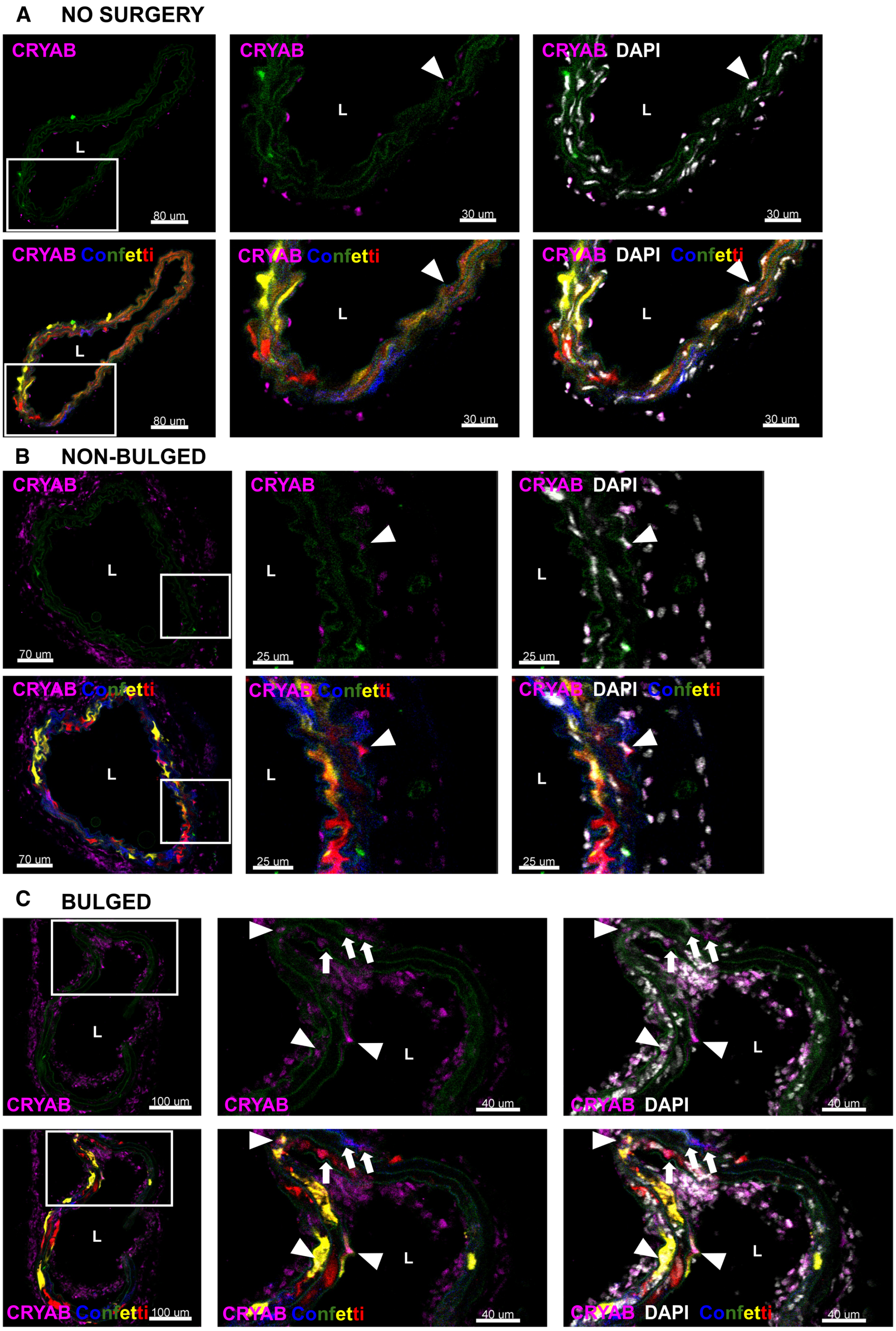

Supplementary Figure XII: Immunostaining for CRYAB. Representative immunofluorescence staining for CRYAB in cryosections from healthy arteries (n=5 animals, average of 4 sections per animal) (**A**) and from either non-bulged (**B**) or bulged (**C**) regions of ligated carotid arteries (day 5-7 post-carotid ligation, n=4 animals, average of 4 non-bulged and 4 bulged sections per animal) from VSMC lineage-labeled Myh11-Confetti animals. The panels at the left show the entire arteries, while panels to the right show

magnified views of the boxed regions. Signals for CRYAB (magenta), Confetti proteins and DAPI (white) are shown as indicated. Arrows point to CRYAB+ cells in patches and arrowheads point to CRYAB+ cells in VSMC singlets. The lumen (L) is indicated. Scale bars are shown on each panel.

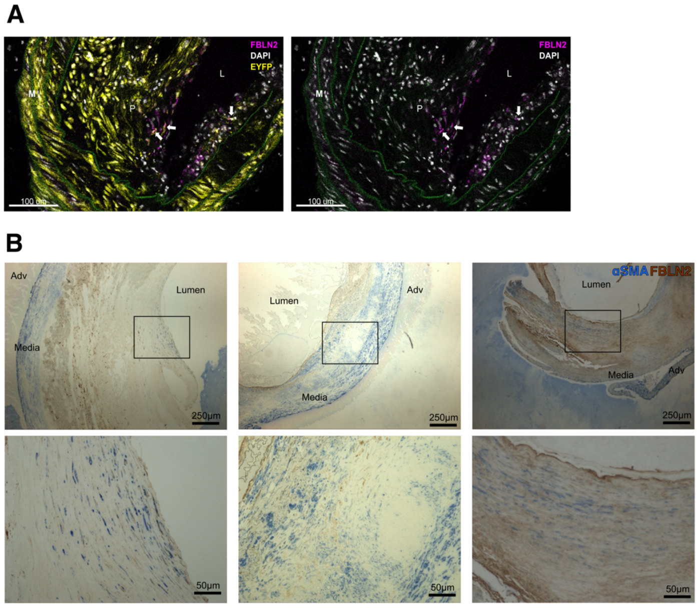

Supplementary Figure XIII: Immunostaining for FBLN2 in atherosclerotic lesions. A, Immunofluorescence staining for FBLN2 (magenta) in arterial cryosections from VSMC-lineage labeled Myh11-EYFP-Apoe animals fed a high fat diet for 24 weeks. The left panel shows a merge with signals for the EYFP VSMC-lineage label (yellow). Green autofluorescence from the elastic laminae outlines the medial layer. Arrows point to EYFP+FBLN2+ cells. P: plaque, M: Medial layer, L: Lumen. Scale bar = 100 µm. **B,** Co-staining for FBLN2 (brown) and αSMA (blue) in FFPE sections from human carotid artery plaques. The adventitia (Adv), media, lumen and plaque region (P) are indicated. Lower panels show magnified views of boxed regions with αSMA+FBLN2+ cells in the plaque region. Representative of 4 patients. Scale bar = 250 µm (top) or 50 µm (lower).

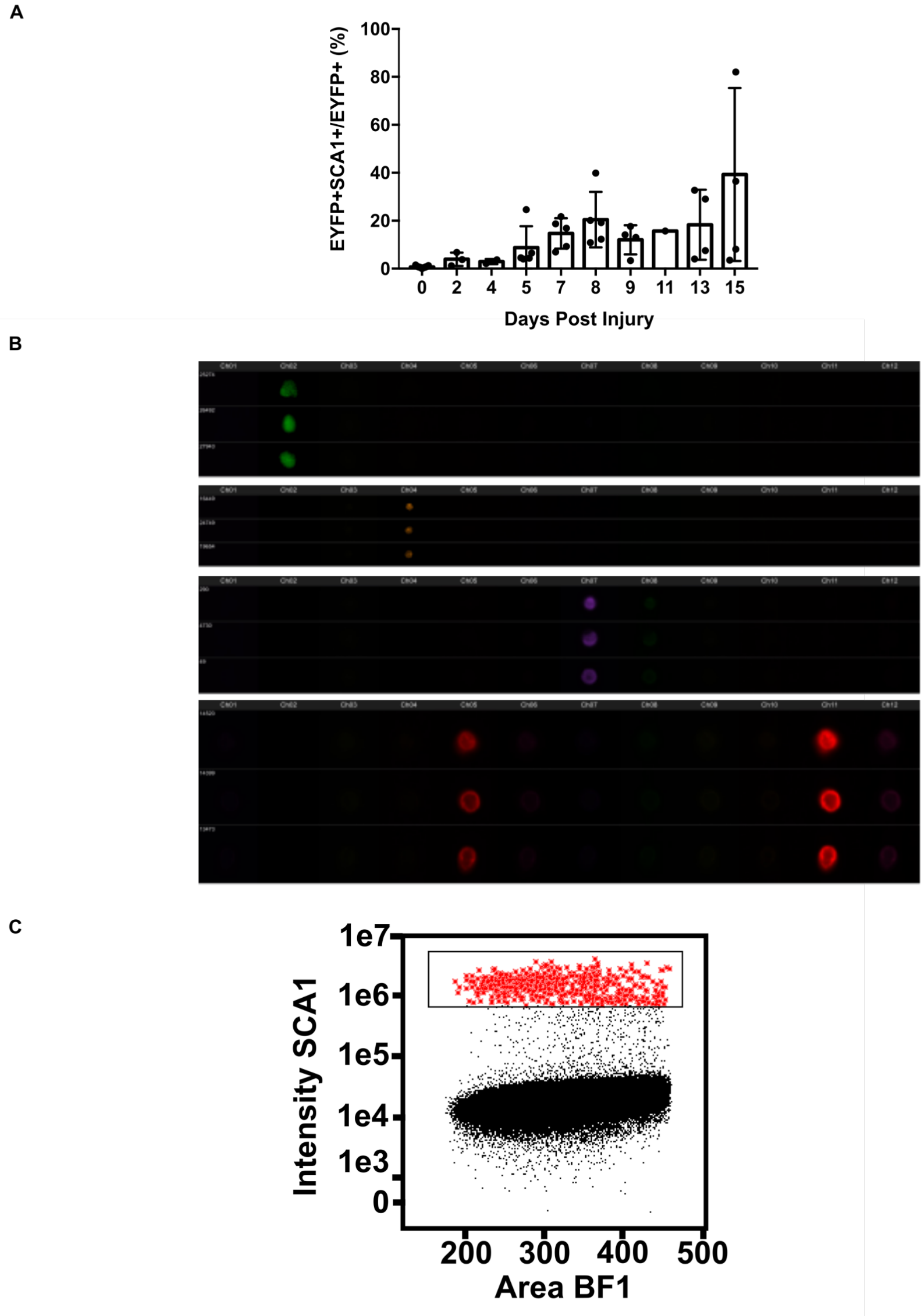

Supplementary Figure XIV: Flow cytometry and ImageStream analysis. A, Percentage of EYFP+ VSMCs expressing SCA1 in left carotid arteries of Myh11-EYFP-KI67/RFP animals analyzed by flow cytometry, showing mean and standard deviation at indicated time points after ligation (total 32). **B,** Controls for ImageStream analysis showing unstained medial VSMCs (EYFP+, top) and adventitial cells (EYFP-) stained with anti-ROCK1 (orange), Phalloidin- iFluor™ 350 (magenta) or anti-SCA1 (red). SCA1 is detected in two channels. **C,** Dotplot showing intensity of SCA1 signal versus bright field area (BF1). Gate used to define SCA1+ cells in Figure 6 is indicated.

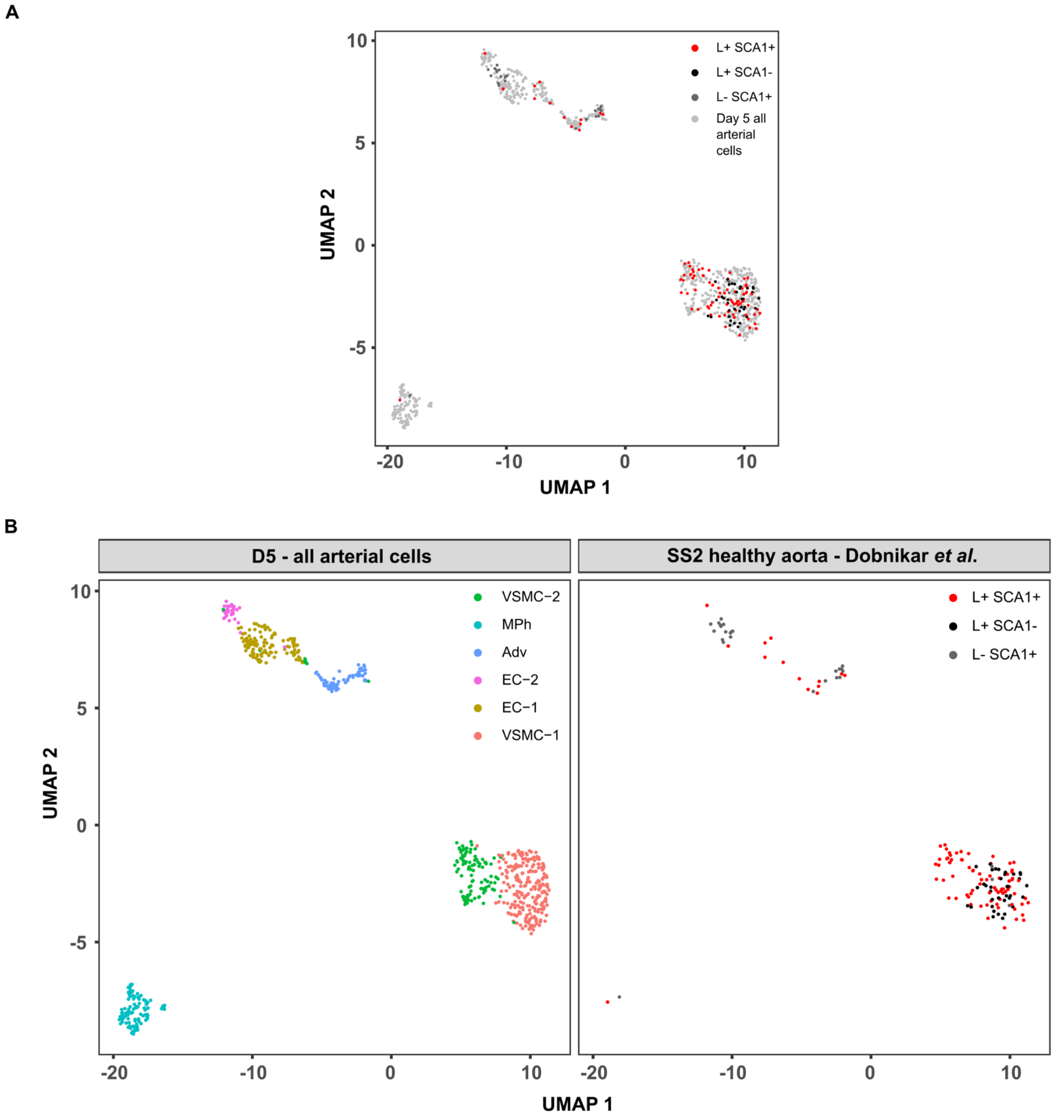

Supplementary Figure XV: SCA1+ VSMCs from healthy arteries are similar to modulated VSMCs following injury. Integration of scRNA-seq dataset of all arterial cells 5 days post carotid ligation (shown in Supplementary Figure VI) with a Smart-seq2 dataset of SCA1+ and SCA1- VSMC-lineage cells from healthy arteries (Dobnikar et al, 2018). In **A,** the UMAP shows integrated data with D5 all arterial cells in grey and VSMC-lineage+ cells (L+) from healthy arteries in red (SCA1+) or black (SCA1-), as well as SCA1+ VSMC-lineage-negative cells in dark grey (L-SCA1+). Please note that lineage-labelling is not 100%, so some VSMC-lineage-negative cells are expected to cluster with VSMCs. In **B**, the integrated data is split by experiment and data for D5 all arterial cells is color-coded by cell population identity (see Supplementary Figure VI and Figure 6C).
