# Supplementary material for: Cellular mechanisms of oligoclonal vascular smooth muscle cell expansion in cardiovascular disease
Source: Cardiovasc Res. 2022 Aug 22;119(5):1279–94. doi: 10.1093/cvr/cvac138 (PMC10202649; doi:10.1093/cvr/cvac138)
Supplement: cvac138_Supplementary_Data [file cvac138_supplementary_data.zip › SupplementaryTable_II_ConfocalMicroscopySettings.docx]

Supplementary Table II: Settings for confocal microscopy

| Sequence Number* | Fluorophore | Excitation Wavelength (nm) | Detection Wavelengths (nm) | Display Colour |
| --- | --- | --- | --- | --- |
| 1 | DAPI | 405 | 410-462 | White |
| 2 | CFP | 458 | 462-543 | Blue |
| 2 | RFP | 555 | 565-632 | Red |
| 3 | YFP | 514 | 520-558 | Yellow |
| 4 | GFP | 488 | 490-507 | Green |
| 4 | Alexa Fluor-647 | 653 | 660-800 | Magenta |

*Sequential scanning was used to avoid spectral overlap.
