# Supplementary material for: Cellular mechanisms of oligoclonal vascular smooth muscle cell expansion in cardiovascular disease
Source: Cardiovasc Res. 2022 Aug 22;119(5):1279–94. doi: 10.1093/cvr/cvac138 (PMC10202649; doi:10.1093/cvr/cvac138)
Supplement: cvac138_Supplementary_Data [file cvac138_supplementary_data.zip › SupplementaryTable_I_Antibodies.docx]

Supplementary Table I: Antibodies used

| **Target** | **Clone/**  **catalogue #** | **Conjugate** | **Host** | **Source** | **Application*** |
| --- | --- | --- | --- | --- | --- |
| αSMA | 1A4/  M0851 |  | Mouse | DAKO | IHC primary (1:400) |
| CD45 | 30-F11/  103124 | Alexa Fluor® 647 | Rat | Biolegend | IF primary (2.5 μg/mL) |
| CRYAB | ab13497 |  | Rabbit | Abcam | IF primary (1 μg/mL) |
| FBLN2 | ab251662 |  | Rabbit | Abcam | IF / IHC primary (1:2000) |
| VCAM1 | MVCAM.A/ 105702 |  | Rat | Biolegend | IF primary (10 μg/mL) |
| SNCG | ab55242 |  | Rabbit | Abcam | IF primary (10 μg/mL) |
| KI67 | 91295 |  | Rabbit | Cell Signaling Technologies | IF mouse primary (0.575 μg/mL) |
| ROCK1 | 21850-1-AP |  | Rabbit | Proteintech | FC/ImageStream primary (3.5 μg/mL) |
| SCA1 | D7 | APC | Mouse | Miltenyi | FC/ImageStream primary (1:10, 3 μg/mL) |
| None (Mouse IgG2a) | MOPC-173/  400203 |  | Rabbit | Biolegend | IHC isotype control (conc. matching primary) |
| None (Rabbit IgG) | ab37415 |  | Rabbit | Abcam | IHC isotype control (conc. matching primary) |
| None (Rat IgG2a) | RTK2758/  400502 | Alexa Fluor® 647 | Rat | Biolegend | IF isotype control (conc. matching primary) |
| None (Rat IgG2b) | RTK4530/  400626 | Alexa Fluor® 647 | Rat | Biolegend | IF isotype control (conc. matching primary) |
| Mouse IgG | E0433 | Biotin | Goat | DAKO | IHC secondary (1:500) |
| Rabbit IgG | A32727 | Alexa Fluor® 555 | Goat | ThermoFisher | FC/Imagestream secondary (1 μg/mL) |
| Rabbit IgG | 8114 | HRP | Goat | Cell Signaling Technologies | IHC secondary (neat) |
| Rabbit IgG | ab150079 | Alexa Fluor® 647 | Goat | Abcam | IF secondary (0.5 μg/mL) |

*IF: immunofluorescent staining, IHC: immunohistochemical staining, FACS: fluorescence activated cell sorting, FC: flow cytometry
